# An amphiphilic dendrimer as a light-activable immunological adjuvant for in situ cancer vaccination

**DOI:** 10.1038/s41467-021-25197-z

**Published:** 2021-08-16

**Authors:** Yongchao Wang, Ningqiang Gong, Chi Ma, Yuxuan Zhang, Hong Tan, Guangchao Qing, Jimei Zhang, Yufei Wang, Jinjin Wang, Shizhu Chen, Xianlei Li, Qiankun Ni, Yuan Yuan, Yaling Gan, Junge Chen, Fangzhou Li, Jinchao Zhang, Caiwen Ou, Yongxiang Zhao, Xiaoxuan Liu, Xing-Jie Liang

**Affiliations:** 1 State Key Laboratory of Natural Medicines and Jiangsu Key Laboratory of Drug Discovery for Metabolic Diseases, Center for Drug Discovery, Center of Advanced Pharmaceuticals and Biomaterials, China Pharmaceutical University, Nanjing, China; 2 Laboratory of Controllable Nanopharmaceuticals, Chinese Academy of Sciences (CAS) Center for Excellence in Nanoscience and CAS Key Laboratory for Biomedical Effects of Nanomaterials and Nanosafety, National Center for Nanoscience and Technology, Beijing, China; 3 University of Chinese Academy of Sciences, Beijing, China; 4 National Center for International Biotargeting Theranostics, Guangxi Key Laboratory of Biotargeting Theranostics, Collaborative Innovation Center for Targeting Tumour Theranostics, Guangxi Medical University, Guangxi, China; 5 Key Laboratory of Medicinal Chemistry and Molecular Diagnosis of the Ministry of Education, College of Chemistry & Environmental Science, Hebei University, Baoding, China; 6 Dongguan Hospital of Southern Medical University, Southern Medical University, Guangdong Provincial Key Laboratory of Shock and Microcirculation, Guangzhou, China

**Keywords:** Tumour immunology, Adjuvants, Cancer immunotherapy

## Abstract

Immunological adjuvants are essential for successful cancer vaccination. However, traditional adjuvants have some limitations, such as lack of controllability and induction of systemic toxicity, which restrict their broad application. Here, we present a light-activable immunological adjuvant (LIA), which is composed of a hypoxia-responsive amphiphilic dendrimer nanoparticle loaded with chlorin e6. Under irradiation with near-infrared light, the LIA not only induces tumour cell lysis and tumour antigen release, but also promotes the structural transformation of 2-nitroimidazole containing dendrimer to 2-aminoimidazole containing dendrimer which can activate dendritic cells via the Toll-like receptor 7-mediated signaling pathway. The LIA efficiently inhibits both primary and abscopal tumour growth and induces strong antigen-specific immune memory effect to prevent tumour metastasis and recurrence in vivo. Furthermore, LIA localizes the immunological adjuvant effect at the tumour site. We demonstrate this light-activable immunological adjuvant offers a safe and potent platform for in situ cancer vaccination.

## Abstract (web summary)

Immunological adjuvants are a crucial component of cancer vaccines. Here the authors design a light-activable immunological adjuvant, based on hypoxia-responsive amphiphilic dendrimer nanoparticles loaded with a photodynamic agent, promoting anti-tumor immune responses in preclinical cancer models.

## Introduction

Cancer immunotherapy, which stimulates the host immune system to recognise and attack tumour cells, has shown great potential for cancer treatment^1–3^. Nanoparticle vaccines that combine adjuvants and tumour antigens in one single platform to induce antigen-specific immune responses are promising strategies for cancer immunotherapy^4,5^. Specifically, in situ preparation of cancer cell lysates-derived cancer vaccine in vivo is a simple and effective method that has attracted great attention for cancer treatment^6,7^. Strategies combining radiotherapy^8–10^, photothermal therapy^11–13^ and photodynamic therapy^14–16^-induced tumour antigen with the encapsulated adjuvants for enhanced in situ vaccination have been developed.

For in situ cancer vaccines, immunological adjuvants (IAs) are essential to elicit sufficient dendritic cells (DCs) activation and evoke the DC-derived co-stimulatory signals required to prime T-cell immune responses^17,18^. Various types of adjuvants have been developed, including mineral salts, microorganism-derived adjuvants and nucleic acid-based adjuvants^19,20^. However, these adjuvants without controllability may easily enter into the circulation and induce severe toxicities which restrict the broad application of cancer vaccines^21,22^. Therefore, an activity controllable adjuvant is an urgent need for spatiotemporal orchestration of antigen presentation and DC activation for efficient in situ vaccination.

**Fig. 1:**
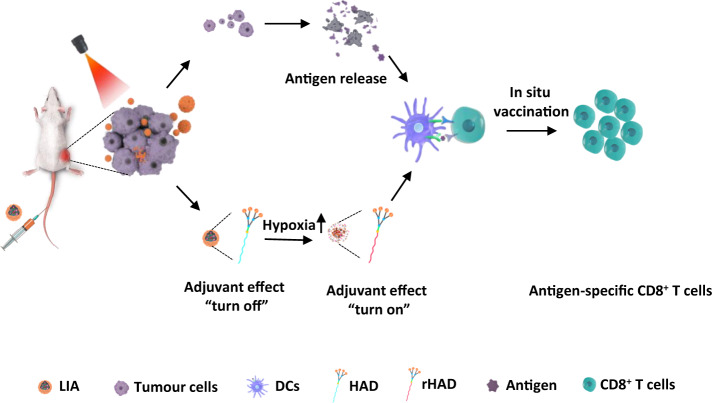
Light-activable immunological adjuvant (LIA) for improved in situ cancer vaccination. The LIA is composed of a hypoxia-responsive amphiphilic dendrimer (HAD) nanoparticle, which is loaded with chlorin e6 (Ce6). After intravenous injection, the LIA accumulates at tumour sites and is then internalized by the cells. Upon near-infrared (NIR) light irradiation, molecular oxygen is rapidly consumed and generates reactive oxygen species (ROS) to induce tumour cell lysis and tumour antigen release. In the meantime, the consumption of oxygen leads to a locally hypoxic microenvironment, which activates the ‘immunological adjuvant’-like effect of the dendrimer. This light-activable immunological adjuvant enhances a robust and safe immune response for in situ cancer vaccination.

Herein, we present a light-activable immunological adjuvant (LIA), composed of a hypoxia-responsive amphiphilic dendrimer (HAD) nanoparticle loaded with a photodynamic agent, chlorin e6 (Ce6). After intravenous injection, the LIA accumulates at the tumour sites and is then internalised by the tumour cells. Upon near-infrared (NIR) light irradiation, molecular oxygen is rapidly consumed and reactive oxygen species (ROS) is generated, which induces cancer cells destruction and tumour antigens release. In the meantime, the hypoxia environment induced by oxygen consumption causes the quick reduction of 2-nitroimidazole groups of the dendrimer to 2-aminoimidazole, which shows an efficient adjuvant effect. The as released tumour antigens combined with the adjuvant (2-aminoimidazole containing dendrimer, rHAD) induce efficient in situ vaccination at the tumour site. The LIA nanoparticles (NPs) that with light-dependent ‘turn on’ in the tumour tissue induce a robust tumour-specific immune response and with reduced systemic toxicity (Fig. 1). The antitumour efficiency of the LIA is evaluated in the breast tumour model and colon tumour model. Moreover, we evaluate the vaccination efficiency in tumour metastasis and recurrence models. All results verify that the light-activable immunological adjuvant mediates a safe and robust in situ vaccination against tumours.

## Result

### LIA preparation and NIR light-induced-nanoparticle transformation

**Fig. 2:**
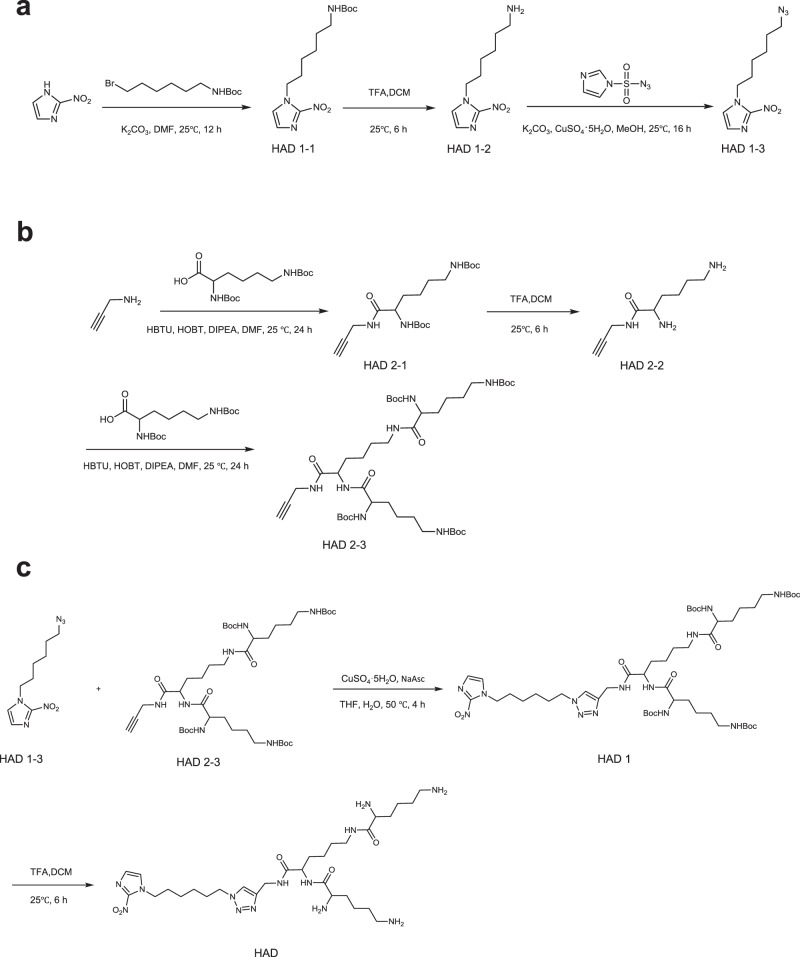
Synthetic route of hypoxia-responsive amphiphilic dendrimer (HAD). **a**–**c** A 2-nitroimidazole derivative and a polylysine dendron were synthesised as the hydrophobic and hydrophilic parts of HAD, then the two parts were conjugated through readily click chemistry to obtain the amphiphilic dendrimer. The synthetic route of the hydrophobic part of HAD (**a**), the synthetic route of the hydrophilic part of HAD (**b**) and the synthetic route of HAD (**c**) were shown.

**Fig. 3:**
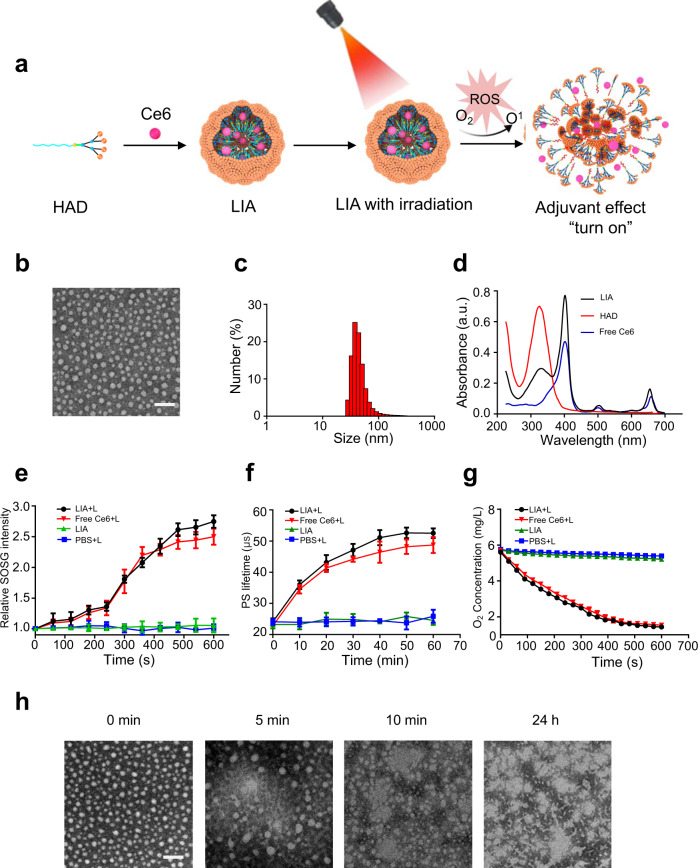
Preparation and characterisation of the LIA. **a** Schematic illustration of the construction of the LIA and its structure is composed of a hypoxia-responsive amphiphilic dendrimer (HAD) nanoparticle loaded with chlorin e6 (Ce6). **b** TEM image of LIA. Scale bar: 100 nm. Experiments were performed three times (*n* = 3) independently, one representative image is shown. **c** DLS size distribution of LIA. Experiments were performed three times (*n* = 3) independently, one representative result is shown. **d** UV-Vis absorbance spectra of free Ce6, HAD and LIA. Experiments were performed three times (*n* = 3) independently, representative results are shown. **e** Generation of singlet oxygen by free Ce6 with laser irradiation (free Ce6+L), LIA without irradiation (LIA) and LIA with irradiation (LIA+L). Generation of singlet oxygen was detected using the singlet oxygen probe SOSG. **f** Induction of hypoxia by free Ce6 and LIA in PBS containing an oxygen-sensitive phosphorescent molecular probe. Increased phosphorescence (PS) lifetime corresponds to decreased oxygen levels. **g** O_2_ concentration of free Ce6 and LIA in PBS using a dissolved oxygen meter. Data in **e**–**g** are presented as mean ± s.d. (*n* = 3) from three independent experiments. **h** TEM images of LIA at different time points. 0 min: before irradiation; 5 min: after irradiation for 5 min; 10 min: after irradiation for 10 min; and 24 h: 24 h after irradiation for 10 min (665 nm, 0.15 W/cm^2^). Scale bar: 100 nm. Experiments in **h** were performed three times independently, representative image for each group is shown.

The detailed synthetic route of the HAD is shown in Fig. 2. A 2-nitroimidazole derivative and a polylysine dendron were used as the hydrophobic and hydrophilic building blocks of HAD, respectively. The amphiphilic dendrimer was synthesised readily using click chemistry^23,24^. The characterisations for each compound are presented in Supplementary Figs. 1–3. Using the film dispersion method^25^, HAD can easily be self-assembled into spherical nanomicelles as evaluated by transmission electron microscopy (TEM) and dynamic light scattering (DLS) (Supplementary Fig. 4). The as-prepared spherical nanomicelles showed a uniform size distribution, with an average diameter of 28.6 ± 2.26 nm and a critical micelle concentration (CMC) of 6.03 μM. To construct the LIA, the photosensitizer Ce6 was encapsulated within the HAD micelles using the film dispersion method (Fig. 3a). The LIA nanoparticle also showed spherical morphology, with an average diameter of 43.22 ± 3.40 nm (Fig. 3b, c). We also demonstrated the successful Ce6 encapsulation in LIA (Fig. 3d). The LIA NPs are stable in both PBS and fetal bovine serum (FBS) containing medium, and limited serum protein adsorption to the nanoparticles was observed after the LIA NPs were incubated with FBS containing medium (Supplementary Fig. 5). The ROS generation ability of LIA was similar to that of free Ce6 (Fig. 3e), investigated using the probe SOSG (singlet oxygen sensor green)^26^. Ce6 was reported to efficiently convert molecular oxygen to singlet oxygen and induced hypoxia. The hypoxia level resulted from NIR laser irradiation of LIA nanoparticle was investigated by measuring the oxygen concentration with an oxygen-sensitive phosphorescent molecular probe^27^. The increased phosphorescence lifetime of the probe in the irradiated samples containing LIA or free Ce6 suggested that the oxygen concentration was decreased compared to the control LIA sample without irradiation (Fig. 3f). The hypoxia following NIR laser irradiation was further confirmed using a dissolved oxygen metre, which showed that the oxygen concentration decreased during the exposure time (Fig. 3g and Supplementary Fig. 6) Subsequently, the light-induced reduction of the 2-nitroimidazole groups and the changes in nanoparticle morphology were investigated in a mimic cell culturing environment containing NADPH and cytochrome *c* reductase^28^. TEM images confirmed the morphological change during the NIR exposure time (Fig. 3h). Furthermore, by analysing the ESI-MS spectra of residues after irradiation, we confirmed that the 2-nitroimidazoles of the dendrimer were gradually reduced to 2-aminoimidazole over the exposure time (Supplementary Fig. 7). These results demonstrate that NIR light irradiation induced effective transformation of 2-nitroimidazole containing dendrimer to 2-aminoimidazole-containing dendrimer.

To evaluate the light-triggered release of Ce6 from LIA NPs, the NPs were loaded in a dialysis bag and were irradiated with NIR light (665 nm, 0.15 W/cm^2^) for different times (5 or 10 min). After 48 h, ~40% of Ce6 was released from LIA NPs with NIR light irradiation for 5 min and the release increased to about 60% in the 10 min NIR light irradiation group. However, only a small amount (< 5%) of Ce6 was released from LIA NPs without NIR light irradiation (Supplementary Fig. 8). These results demonstrated that the hypoxia induced by NIR light irradiation leads to the transformation of 2-nitroimidazole containing dendrimer to 2-aminoimidazole-containing dendrimer, which induced the dissociation of the NPs and the release of Ce6.

### LIA is a NIR light-activable immunological adjuvant

**Fig. 4:**
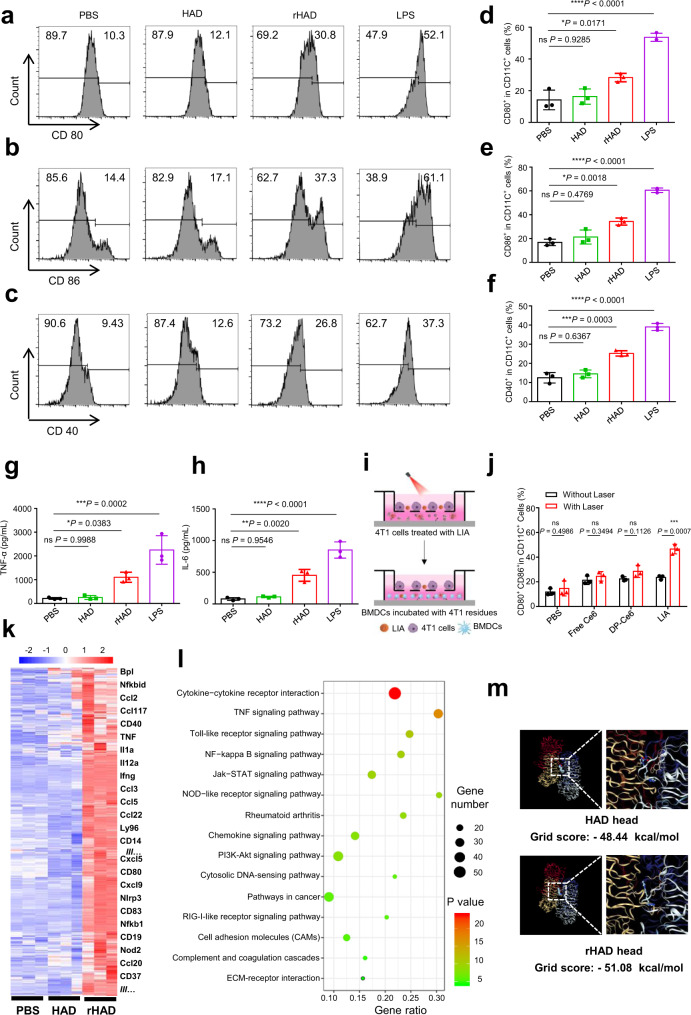
Light-induced activation of immunological adjuvant effect of LIA in vitro. **a**–**f** Flow cytometry analysis of bone marrow-derived dendritic cells (BMDCs) maturation after the cells were incubated with PBS, HAD, rHAD or lipopolysaccharide (LPS). **a**–**c** Flow cytometric histograms of CD80 (**a**), CD86 (**b**) and CD40 (**c**) expressions in BMDCs after different treatments (gated on CD11C^+^ cells). **d**–**f** Quantification of CD80 (**d**), CD86 (**e**) and CD40 (**f**) expressions after BMDCs treated with different groups. **g**, **h** TNF-α (**g**) and IL-6 (**h**) concentrations in BMDCs culture supernatants after 12 h incubation with different formulations (*n* = 3). **i** Schematic illustration of the transwell system experiment. 4T1 tumour cells were first placed in the upper chamber and treated with different formulations, then BMDCs were cultured in the lower chamber. **j** Proportions of CD80^+^ CD86^+^ BMDCs after treated with different treatments (gated on CD11C^+^ cells, *n* = 3). **k**, **l** Transcriptomic analysis of BMDCs after treated with PBS, HAD and rHAD. Three biological replicates are shown. **k** Heat map of DEGs is indicated for each group and Key DEGs are listed on the right. **l** KEGG enrichment analysis of the pathways between rHAD treatment and PBS treatment. **m** Molecular docking of HAD and rHAD head groups with TLR7. Grid score values are calculated from the simulation. Data in **d**–**h** are presented as mean ± s.d. (*n* = 3) and statistically significant differences between groups were identified by one-way ANOVA. Data in **j** are presented as mean ± s.d. (*n* = 3) and statistically significant differences between groups were identified by two-tailed unpaired Student’s *t*-test. ****P < 0.0001, ***P < 0.001, **P < 0.01, *P < 0.05.

In order to investigate the characteristics of the 2-aminoimidazole-containing dendrimer after irradiation, we prepared the reduced version of the dendrimer through reduction with sodium dithionite^29^ (Supplementary Fig. 9). Before testing the nanoparticles in in vitro models, we determined the endotoxin level in these formulations. As shown in Supplementary Fig. 10a, all the formulations are endotoxin-free. We also found that these formulations are non-toxic to normal mammalian cells, NIH3T3 and HUVEC cells (Supplementary Fig. 10b, c). Bone marrow-derived dendritic cells (BMDCs) were utilised to investigate the immunological adjuvant effect of LIA upon irradiation. After BMDCs were incubated with equivalent amounts of HAD or rHAD for 12 h, we observed significant upregulation of the co-stimulatory molecules CD80, CD86 and CD40 in an rHAD-treated group compared with the HAD-treated group (Fig. 4a–f). Cytokine concentrations in the cell supernatant showed that the rHAD-treated group elicited significantly secretion of TNF-α and IL-6 (Fig. 4g, h) compared with the HAD-treated group. These results suggest that the reduced version of the dendrimer can significantly promote the maturation of BMDCs upon irradiation, while the unreduced dendrimer showed a negligible immunological adjuvant effect. To further investigate the interaction of LIA with tumour cells, toxicity and cellular uptake of this nanoparticle in tumour cells were evaluated. As shown in Supplementary Fig. 11, free Ce6 and LIA without irradiation had negligible toxic effects on 4T1 cells, which indicate the biocompatibility of LIA NPs. In contrast, the cell viabilities of 4T1 cells and CT26 cells were drastically decreased in the LIA NPs with 665 nm laser irradiation (2 min, 0.15 W/cm^2^), which suggests that LIA can efficiently kill cancer cells upon NIR light irradiation. Cellular uptake of free Ce6 or LIA by 4T1 cells was observed under confocal laser scanning microscopy (CLSM). As shown in Supplementary Fig. 12, LIA showed enhanced accumulation inside the cells compared to free Ce6. This can be mainly ascribed to the nanoparticle-mediated internalisation of LIA. Additionally, the LIA induced ROS and hypoxia in tumour cells were detected by CLSM in the presence of ROS/hypoxia probes^28^. As shown in Supplementary Fig. 13, the bright green fluorescence represents ROS production (green colour) and hypoxia (red colour) in LIA-treated cells upon NIR irradiation, which indicates that NIR light induced the production of ROS and hypoxic conditions inside the cells. The resulting hypoxic environment inside the cells could reduce the nitroimidazole groups of the dendrimer, thus disrupting the LIA NPs and activating their adjuvant effect. A transwell system was employed to investigate the immunological adjuvant effect of LIA in vitro^16^. In order to investigate if the adjuvant effect is LIA-specific, we also evaluated the activation of BMDCs after free Ce6 or DP-Ce6 (Ce6 encapsulated in DSPE-PEG_2000_) nanoparticle treatment. We found that BMDCs incubated with the LIA-treated tumour cells elicit markedly upregulation of the co-stimulatory molecules after irradiation. In contrast, BMDCs treated with free Ce6 and DP-Ce6 only showed a slight upregulation of the co-stimulatory molecules after irradiation(Fig. 4i, j), which may be due to immunogenic cell death induced by the photodynamic effect^30^.

Next, we used model antigen ovalbumin (OVA) to further verify the immunological adjuvant effect of LIA after irradiation. OT-I CD8^+^ T-cell priming assays were performed in vitro and in vivo^31,32^. As shown in Supplementary Fig. 14a, OT-I cells incubated with BMDCs treated with OVA+rHAD showed comparable interferon (IFN)-γ secretion to the groups treated with OVA+CpG. Meanwhile, the in vivo CD8^+^ T-cell priming assay demonstrated that OVA+rHAD elicit significant proliferation of OVA epitope (SIINFEKL)-specific CD8^+^ T cells (Supplementary Fig. 14b, c). By contrast, OVA+HAD caused a negligible increase in the IFN-γ secretion and proliferation of OVA epitope (SIINFEKL)-specific CD8^+^ T cells. Furthermore, OVA+rHAD showed comparable tumour growth inhibition of B16-OVA tumours with the OVA+CpG group. While OVA+HAD did not provide any significant delay of B16-OVA tumour growth compared with the PBS control group (Supplementary Fig. 14d). These results suggest that rHAD could effectively stimulate BMDCs and elicit subsequent antigen-specific T-cell responses.

To understand the mechanism of the immunological adjuvant effect of LIA after irradiation, we evaluated the transcriptomic features of the BMDCs treated with HAD and rHAD. After treatment with rHAD, 2747 genes were substantially differentially expressed compared with the PBS group (Fig. 4k and Supplementary Fig. 15a, b). Through the Gene Ontology (GO) analysis, most of the differential express genes were found to enrich in the categories of the immune response, defence response and cytokine production (Supplementary Fig. 15c). Genes associated with immune processes were sorted out and marked in Fig. 4k. KEGG enrichment analysis further identified that the significantly expressed genes after rHAD treatment were enriched in immune response-associated signalling pathways and cytokine signalling pathways, such as Toll-like receptor (TLR) signalling pathway and cytokine–cytokine receptor interaction (Fig. 4l). Interestingly, we found the head structure of rHAD was similar to a serial of approved TLR7 agonists which possess the structure of imidazoquinolines^33,34^. This structural similarity inspired us to investigate the interaction of the head groups of rHAD and HAD with TLR7 through dynamic molecular docking^35,36^. Simulated calculation suggested that rHAD head groups showed greater affinity than HAD head groups with the TLR7 (Fig. 4m and Supplementary Table 1). To further confirm that rHAD exerts its effects through the TLR7 signalling pathway, we used a TLR7 inhibitor chloroquine (CQ)^37–39^ to see if it affects rHAD-induced BMDC activation. We found that chloroquine can reverse rHAD-induced BMDC activation (Supplementary Fig. 16a–f). IL-6 and TNF-α production were decreased after chloroquine treatment (Supplementary Fig. 16g, h), which further demonstrate that the rHAD activates the TLR7 pathway in BMDC. Furthermore, chloroquine reserved BMDC activation caused by a classical TLR7 agonist imiquimod (IMQ) (Supplementary Fig. 16a–f) was also observed. Therefore, we demonstrate that the rHAD activates DCs through the TLR7 signalling pathway. Taken together, all these results suggest that the immunological adjuvant of LIA can be controllably activated by NIR light irradiation.

Neoantigen release and antigen-specific immune responses are also crucial for efficient in situ vaccination. A proteomics experiment was performed to examine whether the LIA with NIR light irradiation (LIA+L) induced neoantigen release from tumour cells. 4T1 tumour cells were cultured in an FBS-free medium containing LIA for 4 h and then the cells were irradiated with NIR light for 2 min. After incubating for another 24 h, the supernatant was collected for proteomics analysis. As shown in Supplementary Fig. 17a, several neoantigen peptides^40,41^ were determined from the supernatant of the 4T1 tumour cells after NIR light irradiation. However, no neoantigen peptide was found in the supernatant when the cells were incubated with LIA NPs without NIR light irradiation. These results demonstrate that LIA NPs were internalised by the tumour cells and NIR light irradiation induced neoantigen release from the cells. Next, we explored the antigen presentation of the neoantigens released from tumour cells. We treated the tumour-bearing mice with various nanoparticle formulations, including a PBS control group (PBS, Free Ce6+L, DP-Ce6+L and LIA+L). Splenocytes were isolated from mice euthanized 7 days post-treatment. These splenocytes were co-incubated ex vivo with the supernatant derived from tumour cells that were treated with LIA+L. We found that the splenocytes isolated from the mice treated with LIA+L showed the highest percentage of IFN-γ-secreting CD8^+^ T cells, while other groups showed a limited increase of IFN-γ-secreting CD8^+^ T cells (Supplementary Fig. 17b, c). These results demonstrate that LIA enables efficient tumour neoantigen release and induces a strong neoantigen-specific immune response.

### LIA induced effective antitumour immune responses with limited systemic toxicity

**Fig. 5:**
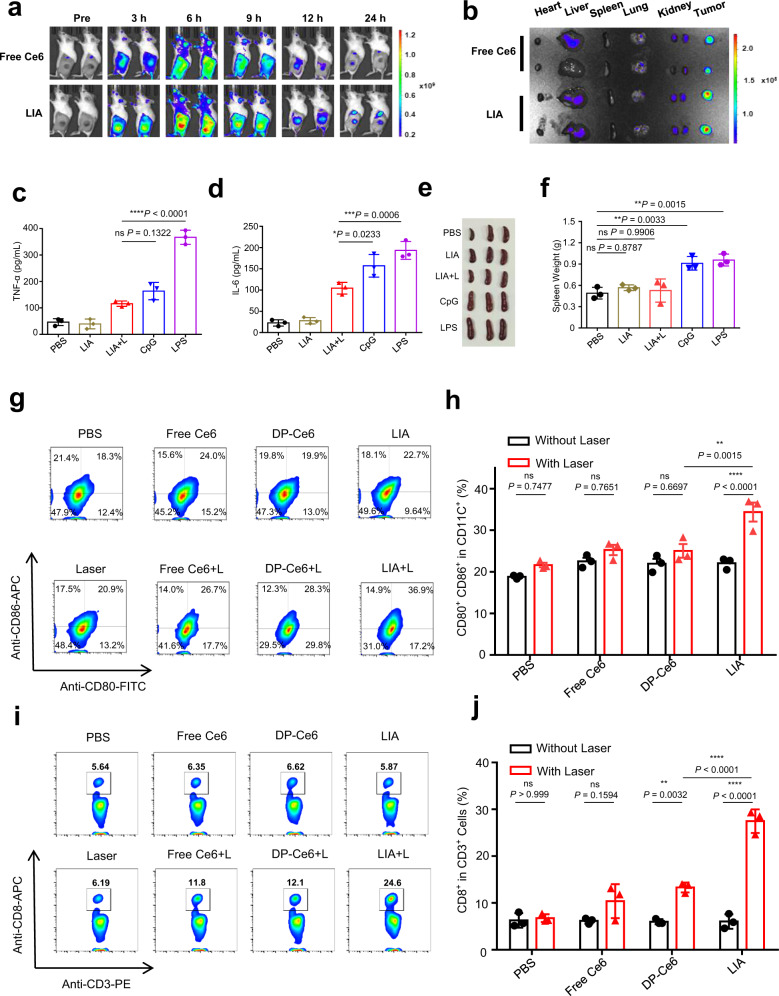
LIA induced reduced systemic toxicity and enhanced immune responses in vivo. **a** Fluorescence images of the 4T1 tumour-bearing mice at 3, 6, 9, 12, 24 h post intravenous administration of free Ce6 and LIA (Ce6 3 mg/kg), 2 mice per group. **b** Fluorescence images of major organs (heart, liver, spleen, lung, kidney) and tumour tissues were collected at 24 h post injection, 2 mice per group. **c**, **d** ELISA analysis of TNF-α (**c**) and IL-6 (**d**) concentrations in serum collected from mice 24 h after different treatments (PBS, LIA, LIA+L, CpG and LPS). **e**, **f** Images of spleens (**e**) and weight of spleens (**f**) from each group 14 days after treatment with different formulations. **g**, **h** The tumour-bearing mice were i.v. injected with PBS, free Ce6, DP-Ce6 and LIA at an equivalent Ce6 dose of 3 mg/kg, then NIR light was used to irradiate (665 nm, 0.15 W/cm^2^, for 30 min) the tumour site at 4 h post injection. The treatments were performed in triplicates every other day. DC maturation was analysed 72 h after the final treatment (gated on CD11C^+^ cells). Representative flow cytometry (**g**) and statistical analysis (**h**) of CD80^+^ CD86^+^ DCs in LNs of mice after different treatments. **i**, **j** Representative flow cytometry (**g**) and statistical analysis (**h**) of CD8^+^ T cells in spleens of mice 7 days after different treatments. Data in **c**, **d**, **f**, **h**, **j** are presented as mean ± s.d. (*n* = 3). Statistically significant differences between groups were identified by one-way ANOVA. ****P < 0.0001, ***P < 0.001, **P < 0.01, *P < 0.05.

We next investigated the biodistribution and tumour accumulation of the LIA NPs in vivo. Firstly, we incubated the nanoparticles with red blood cells and confirm the blood compatibility (Supplementary Fig. 18). Then LIA and free Ce6 were administered intravenously to 4T1 tumour-bearing mice. Compared with the free Ce6 group, a stronger fluorescence signal was detected in the tumour region of LIA-treated mice at 6 h post-injection, and it persisted to 24 h post-injection (Fig. 5a). Imaging of the major organs and tumours demonstrated the long-term retention of LIA in tumours compared with free Ce6 (Fig. 5b). We further conducted an in vivo experiment to explore if the nanoparticle disassembled after intravenous injection. We labelled the LIA NPs with Cy7 and investigated the co-localisation of Ce6 and Cy7. If the LIA NPs disassembled after iv injection, Ce6 may have been released from the nanoparticles and the fluorescence signals from Cy7 and Ce6 should not co-localise. If the LIA NPs remain assembled after iv injection, the fluorescence signals from Cy7 and Ce6 should co-localise. Our results (Supplementary Fig. 19) showed that the fluorescence signals of Cy7 and Ce6 were co-localised at the tumour site at 4, 8 and 12 h post injection, which demonstrates the stability of the LIA NPs in vivo. Given the ability of LIA to accumulate at the tumour site and activation upon irradiation should decrease its systemic toxicities^5,21,22^. We used 4T1 tumour-bearing mice to evaluate the systemic toxicities of LIA. The mice were treated with PBS, LIA, LIA+L, CpG or LPS and the tumour area of the mice in the LIA+L group were exposed to a NIR laser (665 nm, 0.15 W/cm^2^) for 30 min while the other groups were shielded from irradiation. The serum concentrations of cytokines (TNF-α and IL-6) was evaluated at 2 h and 24 h after various treatments. The results revealed that CpG and LPS strongly increased the TNF-α and IL-6 levels in serum at 2 and 24 h post treatment. However, there was a moderate enhancement of the cytokine levels in the LIA+L group (Fig. 5c, d and Supplementary Fig. 20a, b). The body temperature of the mice immunised with LPS was significantly higher 24 h after immunisation, while the temperature rise was negligible in the LIA+L group (Supplementary Fig. 20c). Moreover, significant weight loss was observed in the LPS group especially on days 1 and 2, while the LIA+L group did not show any sign of weight loss (Supplementary Fig. 20d). Spleen swelling was reported to be a sign of acute immunological toxicity^21^. The spleens in the CpG and LPS groups showed significant swelling compared to the PBS, LIA and LIA+L groups (Fig. 5e, f). These results demonstrated that the LIA NPs which can be controllable activated at the tumour site mediated reduced systemic toxicities compared with CpG and LPS.

To verify that the functionalities of light controllable adjuvant activation contribute to their in vivo immune responses, we evaluated the immune stimulation generated by LIA upon irradiation. 4T1 tumour-bearing BALB/c mice were intravenously administered with PBS, free Ce6, DP-Ce6 and LIA, then treated with or without NIR irradiation. Mice treated with LIA showed a significant increase of CD80^+^ CD86^+^ DCs in the inguinal lymph nodes (LNs) 3 days after irradiation. In contrast, mice treated with free Ce6 and DP-Ce6 showed a slight increase of CD80^+^ CD86^+^ DCs in the inguinal lymph node after irradiation (Fig. 5g, h). Moreover, flow cytometric analysis of the proportion of CD8^+^ T cells in the spleen was evaluated 7 days after treatment. Consistent with the increased percentage of mature DCs in draining lymph nodes, the LIA group induced the highest level of CD8^+^ T cell in the spleen after irradiation compared with the control groups (Fig. 5i, j). These results demonstrate that the LIA elicits strong immune responses after irradiation.

### LIA inhibited both primary and abscopal tumour growth in in vivo tumour models

**Fig. 6:**
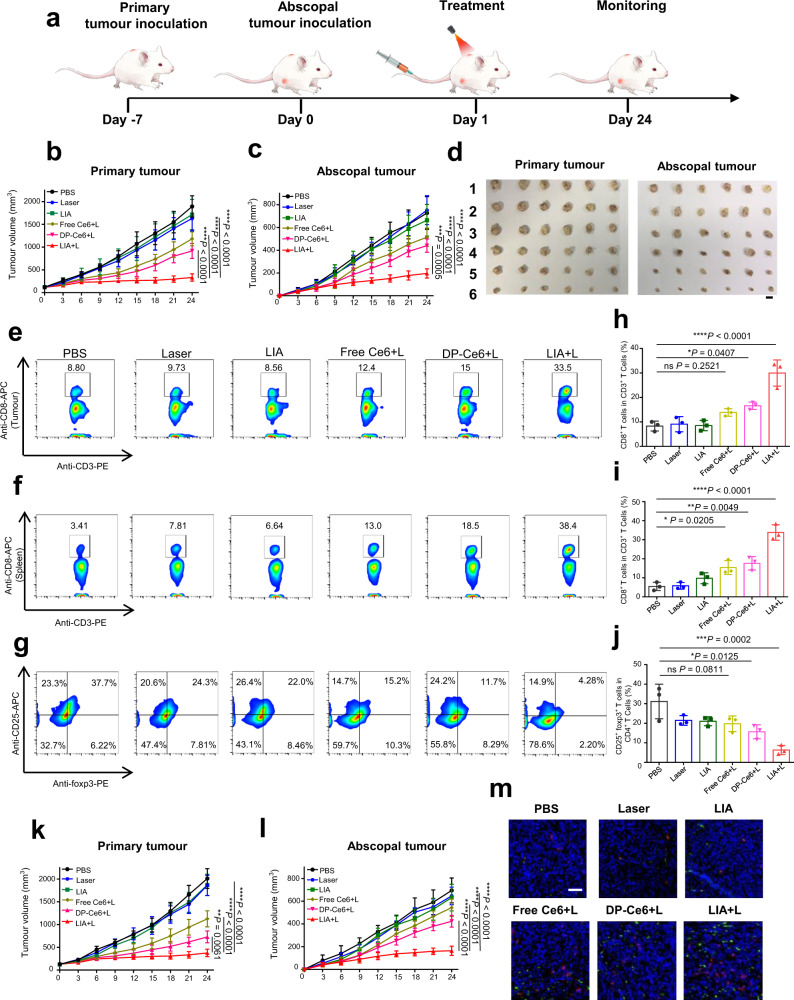
The antitumour effect of LIA to inhibit primary and abscopal tumour growth. **a** Schematic illustration of LIA-mediated antitumour effect to inhibit primary tumour and abscopal tumour growth. **b**, **c** Tumour volumes of 4T1 primary tumours (**b**) and abscopal tumours (**c**) were evaluated (*n* = 8). **d**, Images of the 4T1 primary tumours and abscopal tumours from different groups at day 24. 1, 2, 3, 4, 5 and 6 represent tumour images from mice treated with PBS, Laser, LIA, Free Ce6+L, DP-Ce6+L and LIA+L at an equivalent Ce6 dose of 3 mg/kg, respectively, scale bar is 1 cm. **e**–**j** T-cell infiltration was analysed 7 days after the final treatment. **e**–**g** Flow cytometry data of CD3^+^ CD8^+^ cell proportion in T cells in abscopal tumours (**e**), spleens (**f**) and CD4^+^ CD25^+^ foxp3^+^ cell proportion in CD4^+^ T cells (**g**). **e**–**g** experiments were performed three times independently, representative images are shown. **h**–**j** Statistical analysis of CD3^+^ CD8^+^ cell proportion in T cells in abscopal tumours (**h**), spleens (**i**) and CD4^+^ CD25^+^ foxp3^+^ cell proportion in CD4^+^ T cells (**j**). **k**, **l** Antitumour efficiency tested on CT26 bilateral tumour model. Tumour volumes of CT26 primary tumours (**k**) and abscopal tumours (**l**) were evaluated (*n* = 8). **m** Representative immunofluorescence images of CD8^+^ (green) and CD4^+^ (red) cells infiltrated into abscopal tumours in CT26 tumour model with different treatments. Scale bar, 50 μm. Experiments in **m** were performed three times independently, representative images are shown. Data in **b**, **c** and **k**, **l** are presented as mean ± s.d. (*n* = 8). Data in **h**, **i**, **j** are presented as mean ± s.d. (*n* = 3). Statistically significant differences between groups were identified by one-way ANOVA. ****P < 0.0001, ***P < 0.001, **P < 0.01, *P < 0.05.

Encouraged by the controllable adjuvant activation and efficient immune responses of the LIA NPs after irradiation, we evaluated the antitumour efficacy of LIA in a bilateral breast tumour model. The bilateral breast tumour model was established by inoculating 4T1 cells into the left flank and right flank as indicated in Fig. 6a. The mice were treated by i.v. injection with different formulations at an equivalent Ce6 dose (3 mg/kg) and irradiated the primary tumour with the 665 nm laser (0.15 W/cm^2^, for 30 min) 4 h post injection. The treatments were performed three times every other day. We assessed the antitumour efficacy by measuring the primary and abscopal tumour growth. The mice treated with laser alone showed no inhibition of primary tumour growth and the tumour burden was comparable to the PBS group (Fig. 6b). The moderate anticancer effect was observed in the groups treated with Free Ce6+L and Dp-Ce6+L, which suggests that photodynamic therapy (PDT) can delay tumour growth but cannot inhibit it. The strongest tumour growth inhibition was observed in the LIA+L group. In contrast, no antitumour effect was seen in the LIA without irradiation group, which suggests that the nanoparticles have no antitumour effect in the absence of light irradiation. Consistent with the efficient tumour growth inhibition against a primary tumour, LIA+L significantly suppressed the growth of the abscopal tumour, while the other treatment groups did not show significant suppression of abscopal tumour growth (Fig. 6c, d and Supplementary Fig. 21i, j). To investigate the mechanism of antitumour efficiency of the LIA+L treatment toward abscopal tumours, we evaluated the abscopal tumour-infiltrated T cells by flow cytometry (for gate strategies, see Supplementary Fig. 23). As shown in Fig. 6e, h and Supplementary Fig. 21a, the proportion of CD8^+^ T cells and CD4^+^ T cells infiltrated in abscopal tumours presented a remarkable increase in the LIA+L group compared with the LIA without NIR light irradiation and other control groups. To further confirm the phenotypes of T cells, the numbers of IFN-γ secreting T cells and Treg T cells were also evaluated. The results showed that an increased proportion of IFN-γ secreting T cells (CD8^+^ and CD4^+^ T cells) and a significantly decreased proportion of CD4^+^CD25^+^foxp3^+^ regulatory T cells were observed in abscopal tumours with mice treated with LIA+L (Fig. 6g, j and Supplementary Fig. 21b, d, e). T-cell subsets Th1 and Th17 cells in abscopal tumours were also examined by flow cytometry. As shown in Supplementary Fig. 21b, c, LIA+L induced an improved proportion of Th1 (CD4^+^IFN-γ^+^ T cells), but the proportion of Th17 (CD4^+^IL-17^+^ T cells) cells were not affected when compared with other treatments. The secretion of IFN-γ in the tumour environment was also determined by ELISA assay. Consistent with the results of T-cell infiltration, the secretion of IFN-γ was remarkably enhanced in the mice treated with LIA+L compared with the control groups (Supplementary Fig. 21f). Moreover, immunofluorescence analysis of abscopal tumour sections further confirmed that the tumours in the LIA+L group showed significantly increased CD8^+^ and CD4^+^ T-cell infiltration compared with the control groups (Supplementary Fig. 21g). All these results suggest that the LIA NPs can efficiently destroy the primary tumour and shows an in situ vaccine-like antitumour immune response toward the abscopal tumour.

In addition to the 4T1 tumour model, we further assessed the antitumour efficiency of LIA on the CT26 bilateral tumour model. Consistent with the results of the 4T1 tumour model, mice treated with LIA+L exhibit significant tumour growth inhibition of both primary and abscopal tumour compared with other control groups (Fig. 6k, l). Immunofluorescence analysis and flow cytometry of abscopal tumour indicate a significant increase of CD8^+^ T cells and a decrease of CD4^+^CD25^+^foxp3^+^ regulatory T cells in the LIA+L group (Fig. 6m and Supplementary Fig. 22b–e). Additionally, no significant body weight loss was observed in any of the groups (Supplementary Figs. 21h and 22f). Histology analysis (Supplementary Figs. 24 and 25) did not reveal any observable toxicity in the major organs (heart, liver, spleen, lung and kidney), which suggests the high safety of our formulations. These data further demonstrated that the LIA with irradiation efficiently destroyed the primary tumour and showed an in situ vaccine-like immune response to inhibit the growth of an abscopal tumour.

In the 4T1 model and CT26 models, we used DP-Ce6 as a control group to evaluate the adjuvant effect of the in situ-generated rHAD. However, the Ce6 loading, in vivo biodistribution, photodynamic therapy efficiency and tumour accumulation of these two nanoparticles may be different, which may induce the over-estimate of the adjuvant effect of rHAD. To further confirm the antitumor immune responses that we observed in the LIA+L group are benefited from the in situ-generated rHAD, we systemically compared the Ce6 loading, in vivo biodistribution, photodynamic therapy efficiency and tumour accumulation of these two nanoparticles in vivo. A non-responsive amphiphilic dendrimer (Supplementary Figs. 1 and 3) was also synthesised to encapsulate Ce6 (N-LIA) as a control group. Before we start the animal experiment, Ce6 loading in these nanoparticles was investigated. As shown in Supplementary Table 2, all these three nanoparticles showed similar Ce6 loading level. Then the 4T1 tumour-bearing mice were intravenously injected with N-LIA, DP-Ce6 and LIA at the equivalent dose of Ce6 (3 mg/kg) and the distribution of the nanoparticles was monitored by the IVIS system. As shown in Supplementary Fig. 26a–d, most of N-LIA, DP-Ce6 and LIA NPs were found in the tumour area and the fluorescence signals at 4, 8 and 12 h post-injection were very similar across N-LIA, DP-Ce6 and LIA nanoparticle-treated groups. These results demonstrate that N-LIA, DP-Ce6 and LIA NPs show similar biodistribution and tumour accumulation in tumour-bearing mice. The singlet oxygen generation efficiency of these nanoparticles was also compared, as shown in Supplementary Fig. 26e. N-LIA, DP-Ce6 and LIA NPs showed similar singlet oxygen generation abilities when irradiated with NIR light. The antitumour efficacy of these nanoparticles was evaluated in a bilateral tumour model, where the left flank was defined as the primary tumour and the right flank was defined as the abscopal tumour. N-LIA, DP-Ce6 or LIA was injected intravenously (at the equivalent dose of Ce6 3 mg/kg) and the primary tumour was irradiated with NIR light (0.15 W/cm^2^, for 30 min) at 4 h post injection. The treatments were performed three times at one-day interval. As shown in Supplementary Fig 26f, g N-LIA+L and DP-Ce6+L induced a similar antitumour effect in primary tumours, this demonstrates these two nanoparticles showed similar photodynamic therapy efficiency. However, the abscopal tumour growth was not affected. Surprisingly, LIA+L induced significant tumour growth inhibition in both primary tumours and abscopal tumours compared to N-LIA+L and DP-Ce6+L groups (Supplementary Fig. 26f, g). These results demonstrate that LIA+L may induce a tumour-specific immune response that suppresses abscopal tumour growth. To further investigate if LIA+L induced an antitumour immune response, we depleted the CD8^+^ T cells using the anti-CD8 antibody in the LIA+L group and found that the LIA+L-induced antitumour effect was attenuated in the abscopal tumour (Supplementary Fig 26g). These results demonstrate the LIA+L activated antitumour immune responses in mice. After CD8^+^ T-cell depletion, the primary tumour growth inhibition in LIA+L-treated group was similar to that of DP-Ce6+L and N-LIA+L groups (Supplementary Fig. 26f). These results suggest the LIA+L induced antitumour efficiency contributed by photodynamic therapy was similar to DP-Ce6+L and N-LIA+L groups. Thus, we demonstrate that the activation of tumour-specific immune responses in the LIA+L group leads to significantly improved tumour growth inhibition in vivo.

### LIA elicits a long-term antigen-specific immune memory effect and prevents tumour metastasis in vivo

**Fig. 7:**
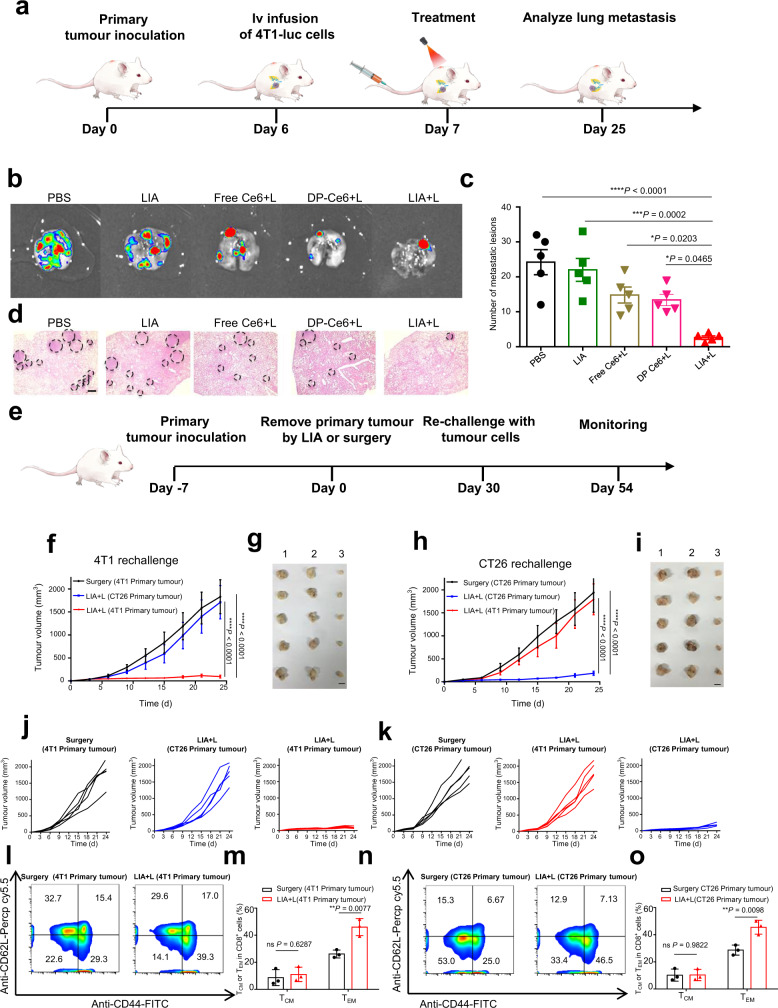
Prevention of tumour metastasis and long-term antigen-specific memory effect. **a** Schematic illustration of treatment schedule for LIA-mediated antimetastatic effect. **b** Bioluminescence images of lung metastasis of i.v injected 4T1-Luc cells in BALB/c mice after various treatments. Experiments were performed five times independently, representative images are shown. **c** Average number of lung metastatic nodules in each group (*n* = 5). **d** Images of H&E staining of lungs are shown. The metastatic nodules are outlined with black circles. Scale bar is 500 µm. Experiments were performed five times independently, representative images are shown. **e** Schematic illustration of treatment schedule for LIA-mediated prevention of tumour recurrence. **f**–**i** Tumour growth curves of s.c. re-challenged 4T1 tumour (**f**) and CT26 tumour (**h**) in BALB/c mice after different treatments with Ce6 dose at the equivalent of 3 mg/kg. Images of tumours in s.c. re-challenged 4T1 tumour (1, 2, 3 represent tumour images from mice treated with surgery (4T1 primary tumour), LIA+L (CT26 primary tumour) and LIA+L (4T1 primary tumour), respectively) (**g**) and CT26 tumour (1, 2, 3 represent tumour images from mice treated with surgery (CT26 primary tumour), LIA+L (4T1 primary tumour) and LIA+L (CT26 primary tumour), respectively) (**i**) in BALB/c mice after different treatments, scale bar is 1 cm. **j**, **k** Individual tumour growth curves of the s.c. re-challenged 4T1 tumour (**j**) and CT26 tumour (**k**). **l**, **n** Representative flow cytometry data of CD3^+^CD8^+^CD44^+^CD62L^+^ (T_CM_) cell or CD3^+^CD8^+^CD44^+^CD62L^−^ (T_EM_) cell percentages in 4T1 tumour (**l**) and CT26 tumour (**n**) model 30 days after different treatments. **m**, **o** Statistical analysis of T_CM_ cell or T_EM_ cell percentages in 4T1 tumour (**m**) and CT26 tumour (**o**) model 30 days after different treatments. Data in **c**, **f**, **h** are presented as mean ± s.d. (*n* = 5). Statistically significant differences between groups were identified by one-way ANOVA. Data in **m**, **o** are presented as mean ± s.d. (*n* = 3). Statistically significant differences between groups were identified by a two-tailed unpaired Student’s *t*-test. ****P < 0.0001, ***P < 0.001, **P < 0.01, *P < 0.05.

Finally, the prevention of metastasis and the long-term immune memory effect of the LIA were evaluated. Tumours were induced by inoculating 4T1 tumour cells into the left flank of mice, and 4T1-luc tumour cells were intravenously injected after the tumour volume reached about 80 mm^3^. Then, the mice were treated with PBS, LIA, Free Ce6+L, DP-Ce6+L or LIA+L, respectively (Fig. 7a). The treatments were performed three times every other day. The lungs were collected to assess metastatic foci at day 25. Mice treated with LIA+L significantly prevented the formation of lung metastases by evaluating the luciferase signals and the average metastatic nodule numbers of the lungs (Fig. 7b, c). Consistent with the bioluminescence imaging, H&E stained lung sections showed fewer lung metastases in the LIA+L group, which further indicates that the lung metastasis was prevented. In contrast, there was no significant protection from the formation of 4T1-luc cells derive lung nodules in control groups (Fig. 7d). We further conduct a tumour cell re-challenge experiment to evaluate the immune memory effect. BALB/c mice bearing 4T1 or CT26 primary tumour in the left flank were treated with the designated procedures (Fig. 7e). Then the mice were re-challenged with the CT26 tumour cells or 4T1 tumour cells into the right flank 30 days post-treatment. The mice treated with surgery did not exhibit any significant inhibition of re-inoculated 4T1 or CT26 tumours. Notably, the mice bearing 4T1 primary tumour with LIA+L treatment completely rejected the re-inoculated 4T1 tumours, while CT26 tumours gradually formed over time (Fig. 7f, g, j). Similarly, mice bearing CT26 primary tumours with LIA+L treatment showed significant growth inhibition of CT26 tumours and negligible influence on the growth of 4T1 tumours (Fig. 7h, i, k). To verify the mechanisms underlying the durable immune responses of LIA, we investigated the memory T cells in the spleens 30 days post-treatment by flow cytometry (for gate strategies, see Supplementary Fig. 27). Mice treated with LIA+L induced a potent memory effect with a significant increase in the frequency of effector-memory T cells (T_EM_ CD3^+^CD8^+^CD44^+^CD62L^−^ or CD3^+^CD4^+^CD44^+^CD62L^−^) compared with the control groups (Fig. 7l–o and Supplementary Fig. 27). These results demonstrated that the LIA can generate sustained antigen-specific immune memory effect to prevent tumour metastasis and recurrence.

## Discussion

In summary, we developed a light-activable immunological adjuvant that spatiotemporally orchestrates DC activation and antigen presentation to function as an in situ vaccine against tumours. NIR laser irradiation at the tumour site induced effective destruction of primary tumours and also induced release of tumour antigens. Moreover, we found that the hypoxia environment induced by the NIR irradiation also led to the transformation of 2-nitroimidazole containing dendrimer to 2-aminoimidazole containing dendrimer, which is an effective immune adjuvant. The rHAD induces dendritic cells maturation via the TLR7 mediated signalling pathway and resulted in boosted antitumour immunity. With the NIR-controllable adjuvant activation, the LIA localises the immunological adjuvant effect at the tumour site and causes reduced systemic toxicity. Our results showed that the LIA mediated an efficient in situ vaccine function which inhibited abscopal tumour growth and prevented tumour metastasis and recurrence. This light-activable immunological adjuvant is highly promising as a controllable delivery platform to improve in situ vaccination.

## Methods

### Materials and reagents

Boc-Lys (Boc)-OH, 1-hydroxybenzotriazole (HOBT) and *N*,*N*,*N*′,*N*′-tetramethyl-*O*-(1H-benzotriazol-1-yl) uronium hexafluorophosphate (HBTU) were obtained from GL Biochem. Ltd. (Shanghai, China). Propargylamine, *N*,*N*-diisopropylethylamine (DIEA), 6-(Boc-amino) hexyl bromide, 2-nitroimidazole and imidazole were obtained from Aladdin Industrial Corporation. chlorin e6 were purchased from J&K Scientific. Ovalbumin (OVA) and lipopolysaccharide (LPS) were purchased from Sigma-Aldrich. CpG oligodeoxynucleotides were purchased from Generay Biotech Co., Ltd. Hydroxychloroquine sulfate was purchased from MedChemExpress (MCE, Shanghai, China), imiquimod was purchased from Topscience (Shanghai, China). ELISA kits for the mouse cytokines TNF-α (catalogue no. 430904), IL-6 (catalogue no. 43304) and IFN-γ (catalogue no. 430804) were obtained from Biolegend. Anti-mouse antibodies anti-CD80-FITC (catalogue no. 104705), anti-CD86-APC (catalogue no. 105113), anti-CD11c-PE (catalogue no. 117307), anti-CD3-PE (catalogue no. 100312), anti-CD40-APC (catalogue no. 124611), anti-CD4-FITC (catalogue no. 100510), anti-CD4-PE (catalogue no. 100408), anti-foxp3-PE (catalogue no. 126403), anti-CD25-APC (catalogue no. 101909), anti-CD8-APC (catalogue no. 100712), anti-CD62L-PerCP/Cyanine5.5 (catalogue no. 104431), anti-CD44-FITC (catalogue no. 103006), anti-CD62L-APC (catalogue no. 104412), anti-CD44-PE (catalogue no. 103007), anti-mouse IFN-γ- FITC (catalogue no. 505806), anti-mouse IL-17A- APC (catalogue no. 506915), Cell Activation Cocktail (catalogue no. 423303), Ultra-LEAF™ Purified anti-mouse CD8a (catalogue no. 100746) were purchased from Biolegend. PE-conjugated H-2Kb/OVA (SIINFEKL) tetramer (catalogue no. TB-5001–1) was purchased from Medical and Biological Laboratories Co. Ltd. Antibodies were diluted 200 times for the flow cytometry experiments. Recombinant mouse IL-4(catalogue no. Z02996-50) and recombinant mouse GM-CSF (catalogue no. Z02979-10) were obtained from GenScript Biotech Corporation. Collagenase type IV (catalogue no. C8160) and CCK-8 cytotoxicity assay kit (catalogue no. CA1210) were obtained from Solarbio. 0.25% trypsin-EDTA, Hoechst 33342 and antibiotic solution were obtained from Life Technologies.

### Cell culture

4T1 cells, CT26 cells, NIH3T3 cells and HUVECs were purchased from the Institute of Basic Medical Sciences, Chinese Academy of Medical Sciences. 4T1 Cells and CT26 cells were cultured in RPMI-1640 medium containing 10% FBS and 1% penicillin/streptomycin at 37 °C with 5% CO_2_. NIH3T3 cells, HUVECs and B16F10-OVA cells (a gift from C. Xu.) were cultured in DMEM medium containing 10% FBS and 1% penicillin/streptomycin at 37 °C with 5% CO_2_. All the cell lines authentication was conducted by a short tandem repeat DNA profiling and confirmed the cells were not contaminated by mycoplasma.

### Animals

BALB/c mice and C57BL/6 mice (6–8-weeks-old) were purchased from Vital River Laboratory Animal Technology Co. Ltd. OT-I transgenic mice were a gift from M. Zhu, Institute of Biophysics, Chinese Academy of Sciences. Mice were maintained in a 12 h light-dark cycle at 25 °C, 40% relative humidity with free access to food and water. All animal experiment protocols were approved by the Institutional Animal Care and Use Committee of the National Center for Nanoscience and Technology.

### Synthesis of HAD 1-1

Briefly, 100 mg 2-nitroimidazole (0.880 mmol) and 185 mg K_2_CO_3_ (1.34 mmol) were added in dimethylformamide (DMF) and stirred at 25 °C for 1 h. Then, 267 mg 6-(Boc-amino) hexyl bromide (0.950 mmol) was added into the mixture. The mixture was stirred for 12 h. The solvent was removed to give the product. The product was extracted with ethyl acetate by dissolving in water. The ethyl acetate with the product was dried with anhydrous Na_2_SO_4_. The product was isolated with column chromatography (yield: 85%).

### Synthesis of HAD 1-2

HAD 1-1 was dissolved in dichloromethane (DCM). Then trifluoroacetic acid (TFA, 10.0 equiv, according to the number of Boc groups) was added into the solution under ice-bath and stirred at 25 °C for 6 h to remove Boc groups and precipitated with anhydrous diethyl ether to finally obtain the white solid product (yield: 89%).

### Synthesis of imidazole-1-sulfonyl azide

Imidazole-1-sulfonyl Azide was obtained following a method described previously^42^. NaN_3_ (320 mg, 4.92 mmol) was added to ice-cold acetonitrile (10.0 mL). Sulfuryl chloride (0.400 mL, 4.92 mmol) was added. After 12 h, imidazole (680 mg, 10.0 mmol) was introduced to the mixture (ice-cooled) over 30 min and the reaction was kept at 25 °C for 6 h. The mixture was washed by DI H_2_O, saturated aqueous NaHCO_3_. The solution was dried using anhydrous Na_2_SO_4_ and the mixture was then purified with column chromatography to give the product (yield: 52%).

### Synthesis of HAD 1-3

6-(2-nitroimidazole) hexylamine (106 mg, 0.500 mmol), K_2_CO_3_ (138 mg, 1.00 mmol) and CuSO_4_.5H_2_O (2.50 mg, 0.01 mmol) were added in 5.00 mL MeOH under a N_2_ atmosphere. Imidazole-1-sulfonyl azide (173 mg, 1.00 mmol) was added dropwise into the solution and kept at 25 °C for 16 h. Then the solution was washed by saturated aqueous NaCl and purified to obtain the product (yield: 68%).

### Synthesis of HAD 2-1

Propargylamine (53.0 mg, 0.960 mmol), Boc-Lys(Boc)-OH (398 mg, 1.15 mmol), 509 mg HBTU (1.34 mmol) and 183 mg HOBT (1.34 mmol) were dissolved in anhydrous dimethylformamide (DMF) under an N_2_ atmosphere. N, N-diisopropylethylamine (DIPEA) (619 mg, 4.80 mmol) was added to the above mixture. Then the mixture was kept at 25 °C for 24 h. The solution was washed three times each with saturated NaHCO_3_, HCl (1 M) and saturated NaCl. The liquid was concentrated and purified with column chromatography to give the product (yield: 85%).

### Synthesis of HAD 2-2

HAD 2-1 was dissolved in 5.00 mL dichloromethane. Then trifluoroacetic acid (TFA, 10.0 equiv, according to the number of Boc groups) was added dropwise into the liquid at 0 °C. Then the mixture was kept at 25 °C for 6 h to remove Boc groups. The solution was evaporated to remove DCM and TFA, then precipitated with anhydrous diethyl ether to give the white product (yield: 93%).

### Synthesis of HAD 2-3

Boc-Lys (Boc)-OH (415 mg, 1.20 mmol), HAD 2-2 (205 mg, 0.500 mmol), 531 mg HBTU (1.40 mmol) and 190 mg HOBT (1.40 mmol) were dissolved in anhydrous dimethylformamide (DMF) under an N_2_ atmosphere. *N*,*N*-diisopropylethylamine (DIPEA) (645 mg, 5.00 mmol) was added to the above mixture. Then the solution was kept at 25 °C for 24 h. The mixture was washed three times each with saturated NaHCO_3_, HCl (1 M) and saturated NaCl. The liquid was concentrated and purified with column chromatography to give the product (yield: 82%).

### Synthesis of HAD 1

HAD 1 was synthesised according to a method described previously^43,44^. HAD 1-3 (40.0 mg, 0.168 mmol), CuSO_4_·5H_2_O (4.0 mg, 0.016 mmol) and NaAsc (6.90 mg, 0.035 mmol) were dissolved into 3.00 mL THF under a N_2_ atmosphere. 1.00 mL THF containing 141 mg HAD 2-3 (0.168 mmol) was added to the solution. Subsequently, 1.00 mL H_2_O was added into the mixture and kept at 50 °C and shaded from light for 4 h. The mixture was washed with EDTA-2Na solution, dried by anhydrous Na_2_SO_4_ and purified with column chromatography to give the product (yield: 72%).

### Synthesis of HAD

HAD 1 was suspended in DCM. Then TFA was added into the solution at 0 °C and the mixture was kept at 25 °C for 6 h to remove Boc groups. The mixture was precipitated with anhydrous diethyl ether to give a white solid product (yield: 92%).

### Synthesis of rHAD

Sodium dithionite (261 mg, 1.50 mmol) was dissolved into a solution of HAD (677 mg, 1.00 mmol). The reaction solution was kept under a nitrogen atmosphere for 24 h. Then the solution was dialysed against water and lyophilised to obtain the product.

### Synthesis of N-HAD 1-1

1,6-dibromohexane (2.25 g, 9.23 mmol) was dissolved in anhydrous DMF (15.0 mL), to which sodium azide (200 mg, 3.08 mmol) was added in portions during 25 min. The mixture was stirred overnight at 65 °C in the dark. The solvent was then removed under vacuum before distilled water was added. The aqueous layer was extracted with ethyl acetate and then the organic layers were combined, dried over Na_2_SO_4_ and concentrated. The crude was purified by column chromatography to give the product as a colourless oil (yield: 89%).

### Synthesis of N-HAD 1-2

Briefly, tetrahydropyrrole (138 mg, 1.94 mmol) and K_2_CO_3_ (268 mg, 1.94 mmol) were added in dimethylformamide (4.00 mL) and stirred at 25 °C for 1 h. Then, N-HAD 1-1 (200 mg, 0.97 mmol) was added into the mixture. The mixture was stirred for 12 h. The solvent was removed to give the product. The product was extracted with ethyl acetate by dissolving in water. The ethyl acetate with the product was dried with anhydrous Na_2_SO_4_. The product was isolated with column chromatography (yield: 75%).

### Synthesis of N-HAD 1

N-HAD 1-2 (40.0 mg, 0.200 mmol), CuSO_4_·5H_2_O (5.0 mg, 0.020 mmol) and NaAsc (8.0 mg, 0.040 mmol) were dissolved into 3.00 mL THF under a N_2_ atmosphere. 1.00 mL THF containing HAD 2-3 (205 mg, 0.24 mmol) was added to the solution. Subsequently, 1.00 mL H_2_O was added into the mixture and kept at 50 °C and shaded from light for 4 h. The mixture was washed with EDTA-2Na solution, dried by anhydrous Na_2_SO_4_ and purified with column chromatography to give the product (yield: 70%).

### Synthesis of N-HAD

N-HAD 1 was suspended in DCM. Then TFA was added into the solution at 0 °C and the mixture was kept at 25 °C for 6 h to remove Boc groups. The mixture was precipitated with anhydrous diethyl ether to give a white solid product (yield: 93%). The as-obtained product was characterised using ^1^H-nuclear magnetic resonance spectroscopy (^1^H-NMR) (with MestReNova 9.0 software).

### Preparation and characterisation of LIA NPs

The nanomicelles were prepared through a film dispersion method^25^. Briefly, HAD and Ce6 at mass ratios (5:1) were dissolved in 5.00 mL mixed solvent (chloroform: methanol = 3:2, vol/vol). The film was formed through a rotary evaporator under reduced pressure, then hydrated with PBS at 60 °C for 40 min under stirring. The encapsulation efficiency (EE) and drug-loading efficiency (DL) were determined by measuring the UV-Vis absorbance of Ce6 after the disruption of the nanoparticles in methanol. The encapsulation efficiency was calculated as EE = *W*_e_/*W*_O_ × 100%, DL = *W*_e_/*W*_L_ × 100%, *W*_e_ represent the Ce6 encapsulated in the LIA NPs, *W*_O_ represent the initial amount of Ce6 during the preparation, *W*_*L*_ represent the total weight of nanoparticles after lyophilization. The particles were stained with 2% phosphotungstic acid and the morphology was observed using TEM (HT7700, HITACHI, Japan) or Tecnai G2 F20 (FEI, USA) system with digital micrograph3.7 software. The size and polydispersity were determined by dynamic light scattering (DLS, Zetasizer nano SZ90, Malvern, UK).

### Critical micelle concentration of HAD

Pyrene was used to detect the critical micelle concentration of HAD. HAD solutions with concentrations ranging from 1.2 × 10^−7^ to 5.0 × 10^−4^ mol/L were prepared. Then the solutions were added to flasks containing pyrene, at the concentration of 6.0 × 10^−7^ mol/L and sonicated for 30 min. After that, the solutions were kept for 2 h (RT) to promote micelle formation. Fluorescence spectra were measured by a fluorescence spectrophotometer (F-7000; HITACHI), the excitation wavelength was 335 nm. The fluorescence intensity ratio of I373/I384 was analysed as a function of the logarithm of HAD concentration.

### LIA-mediated generation of singlet oxygen in vitro

Solutions of free Ce6, N-LIA, DP-Ce6 and LIA with an equivalent Ce6 concentration (10 μg/mL) in phosphate-buffered solutions (PBS) were mixed with the probe singlet oxygen sensor green (SOSG)^26^. The concentration of SOSG was 2.5 μM. The solutions were irradiated with a laser (665 nm, 0.15 W/cm^2^). The relative fluorescence intensity was recorded at a predetermined time using a fluorescence spectrophotometer (F-7000; HITACHI).

### LIA-mediated oxygen consumption in vitro

The MitoXpress Kit (Cayman Chemical) and a dissolved oxygen meter were used to evaluate the oxygen consumption rate under irradiation^45^. Briefly, 150 μL of LIA (10 μg/mL Ce6 concentration) and 100 μM DPBF was added in a 96-well plate and covered with oil. The samples were treated with irradiation (665 nm, 0.15 W/cm^2^) for different time intervals. Then MitoXpress probe (10 μL) was added into the samples. The time-resolved fluorescence (TR-F) intensity was analysed using a microplate reader with excitation and emission wavelength set as 380 and 600 nm, respectively. The results were used to calculate the phosphorescence lifetime. PBS and LIA without irradiation were examined as controls. O_2_ concentration was further evaluated by a dissolved oxygen meter after the samples were exposed to irradiation at different times. Briefly, LIA (10 μg/mL Ce6 concentration) in PBS was irradiated (665 nm, 0.15 W/cm^2^) for different times. After that, the O_2_ concentration was detected by a dissolved oxygen meter during the exposure time. PBS and LIA without irradiation were used as controls. LIA with different Ce6 concentrations was also evaluated by the same method.

### Light-induced morphology change and group transformation

To evaluate the responsiveness of LIA to hypoxia, freshly prepared LIA (20 μg/mL Ce6 concentration) was incubated in PBS containing 20 μM NADPH and 5 μg/mL cytochrome *c* reductase and covered with oil. The samples were irradiated with laser (665 nm, 0.15 W/cm^2^) for predetermined times. The morphology of LIA NPs at different exposure times were observed by TEM (HT7700, HITACHI, Japan), the samples were stained with 2% phosphotungstic acid for 2 min before observation. After different exposure times, the samples were analysed by ESI-MS to confirm the reduction of the nitroimidazole groups.

### In vitro Ce6 release from the LIA NPs

400 μL of 1 mg/mL LIA NPs with 100 μM NADPH and 5 μg/mL cytochrome *c* reductase in PBS buffer were added into a dialysis tube (10 K MWCO) embedded into 3.5 mL of PBS buffer and gently shaken at 37 °C, 200 rpm. The samples were exposed to laser (665 nm, 0.15 W/cm^2^) for 5 min or 10 min. The samples without laser irradiation were set as controls. The release medium (200 μL) was sampled at predetermined time points, and the same volume of fresh medium was supplemented. Ce6 in the release medium was determined by measuring the UV-Vis absorbance of Ce6.

### Isolation of bone marrow-derived dendritic cells (BMDCs)

BMDCs were obtained from the femurs and tibiae of C57BL/6 mice(6–8 weeks old) following a previously reported method^46^. Bone marrow cells were cultured in RPMI-1640 medium contained 10% FBS and 1% penicillin/streptomycin at 37 °C containing 5% CO_2_. 10 ng/mL murine recombinant mouse granulocyte/macrophage colony-stimulating factor and 50 ng/mL IL-4 were added and the medium was half replaced every other day.

### Cellular uptake and distribution

For the intracellular distribution study, 4T1 cells were cultured with free Ce6 and LIA (5 μg/mL Ce6 concentration) for different incubation times (1, 2, 4 and 8 h). At the predetermined time point, the samples were washed with PBS, then treated with the nuclear stain Hoechst 33342 for 20 min and imaged by a laser scanning confocal fluorescence microscope (ZEISS, LSM710 with ZEN 2010 software).

### Cytotoxicity assay

NIH3T3 cells and HUVEC cells were incubated with HAD and rHAD with the concentration ranging from 0 to 100 μg/mL for 24 h. Then the cell viability was evaluated by CCK-8 cytotoxicity assay kit. 4T1 cells and CT26 cells were incubated with free Ce6 and LIA of different concentrations. Then the cells were treated with irradiation for 2 min (665 nm, 0.15 W/cm^2^) or shielded from light 4 h post incubation and the cell viability was evaluated by CCK-8 cytotoxicity assay kit.

### LIA-mediated generation of ROS and hypoxia in tumour cells

To evaluate the ROS/hypoxia status of cells exposed to LIA under irradiation, a hypoxia/oxidative detection kit (Enzo Life Sciences) was used as an indicator^45^. After incubated with LIA (Ce6 concentration 2.5 μg/mL) for 4 h, the hypoxia/oxidative detection mixture was added and cultured for 30 min. After being treated with irradiation for 2 min (665 nm, 0.15 W/cm^2^), the cells were washed and observed by a confocal laser scanning microscope (UltraVIEW VoX with Volocity software 6.1.1).

### Analysis of LIA-mediated DC maturation upon irradiation in vitro

For flow cytometry analysis, immature BMDCs were treated with PBS, rHAD (10 μg/mL) and HAD (10 μg/mL) for 12 h. After that, BMDCs were collected and stained with antibodies and analysed by flow cytometry. Meanwhile, the IL-6 and TNF-α concentrations in the culture supernatant were determined using ELISA kits. Alternatively, immature BMDCs were co-incubated with the residues of 4T1 tumour cells using a transwell system. The 4T1 cells were incubated with PBS, free Ce6, DP-Ce6, or LIA (Ce6 concentration 2.5 μg/mL) with or without irradiation (665 nm, 0.15 W/cm^2^, 2 min). Then BMDCs were incubated with the residues of 4T1 cells for 12 h. The cells were stained with antibodies and analysed by flow cytometry (BD Accuri C6 1.0.264.21, BD, USA).

### Transcriptomics assay

BMDCs isolated from C57BL/6 mice were treated with PBS, rHAD (10 μg/mL) and HAD (10 μg/mL). After incubation for 12 h, BMDCs were collected and the sequencing was performed in Berry Genomics Co., Ltd. DEGSeq R package (1.20.0) was used to analyse the differentially expressed genes. The Benjamini & Hochberg method was used to adjust the *P*-values. Significantly differential expressed genes were determined by setting the threshold of corrected *P*-value of 0.05 and log2(Fold change) of 1.

### Molecule-docking assay

Dynamic molecular docking was performed on Yinfo Cloud Platform (https://cloud.yinfotek.com/) by the Dock 6 protocol. The crystal structure of Toll-like receptor protein7 (PDB code: 5mgh, resolution: 2.20 Å) was downloaded from the RCSB Protein Data Bank. The head structure of HAD and rHAD were processed to a 3D structure with energy minimisation in MMFF94 force field. The crystal ligand was used to define the binding pocket. Semi-flexible docking and output poses were performed by DOCK 6.7 programme and calculated by the Grid scoring function.

### CD8^+^ T-cell priming assay in vitro

To further verify the immunological adjuvant effect of LIA after irradiation, the IFN-γ secretion of OT-I T cells primed by BMDCs pulsed with different treatments were analysed. Briefly, BMDCs were cultured with 2.5 µg/mL OVA, or OVA mixed with HAD, rHAD and CpG at 37 °C for 12 h. These BMDCs were then co-cultured with CD8^+^ T-lymphocytes collected from spleen cell suspensions of OT-I mice. After incubation for another 12 h, the collected supernatant was used for IFN-γ detection.

### CD8^+^ T-cell priming assay in vivo

C57BL/6 mice were intravenously injected with 5 × 10^4^ CD8^+^ T cells collected from spleen cell suspensions of OT-I mice. Then the mice were subcutaneously immunised with OVA (10 μg) or OVA mixed with HAD (50 μg), rHAD (50 μg) and CpG (50 μg) at the tail base three times a week for 3 weeks. One week after the final injection, spleens were isolated and single-cell suspensions of the splenocyte were obtained. The cells were stained with anti-CD8-APC, Anti-H-2Kb/SIINFEKL-PE before flow cytometry analysis.

### Tumour inhibition of B16-OVA tumour

C57BL/6 mice (6–8 weeks) were subcutaneously inoculated with 1 × 10^6^ B16-OVA cells (day 0). Mice were subcutaneously immunised with OVA (10 μg) or OVA mixed with HAD (50 μg), rHAD (50 μg) and CpG (50 μg) at the tail base (days 5 and 12). Tumour size was measured and calculated as *V* = length × width^2^/2. When the length or width of the tumours was larger than 20 mm, or when the bodyweight loss reached 20%, the mice were euthanized.

### DC activation mechanism study

For flow cytometry analysis, immature BMDCs were treated with PBS, rHAD (10 μg/mL), HAD (10 μg/mL) and imiquimod (2 μg/mL) for 12 h. For the inhibition study, immature BMDCs were pre-treated with chloroquine (5 μg/mL) for 1 h, then incubated with rHAD (10 μg/mL) and imiquimod (2 μg/mL) for 12 h. After that, BMDCs were collected and stained with antibodies and analysed by flow cytometry. Meanwhile, the IL-6 and TNF-α concentrations in the culture supernatant were determined using ELISA kits.

### Detection of antigen release and antigen-specific immune responses

4T1 tumour cells were incubated in an FBS-free medium containing LIA (Ce6 concentration 5 μg/mL) for 4 h Then the cells were treated with irradiation (665 nm, 0.15 W/cm^2^) for 2 min and incubated for another 24 h. The supernatant was collected and sent to Beijing Qinglian Biotech Co., Ltd for proteomics assay. Neoantigen peptides previously reported^40^ were identified by nano-liquid chromatography-electrospray ionisation-tandem mass spectrometry (nano-LC/ESI-MS/MS). For the antigen-specific immune responses assay, 4T1 tumour-bearing mice were intravenously injected with Free Ce6, DP-Ce6 and LIA at the equivalent dose of Ce6 (3 mg/kg), then NIR light (665 nm, 0.15 W/cm^2^, for 30 min) was used to irradiate the tumour site 4 h post injection. The treatments were performed in triplicates every other day. 7 days after the final treatment, 2 × 10^5^ splenocytes isolated from the mice were stimulated with the supernatant of tumour cells treated with LIA after irradiation for 24 h. After stimulation, the splenocytes were collected and analysed by flow cytometry.

### Evaluation of systemic toxicity

4T1 tumour-bearing BALB/c mice were intravenously administrated with PBS, LIA (3 mg/kg of Ce6, with or without laser irradiation), subcutaneously injected with CpG (50 ug per mouse) or LPS (10 µg per mice) at the tail base. After 4 h, the tumour in LIA+laser group was irradiated with NIR laser (665 nm, 0.15 mW/cm^2^) for 30 min. Bodyweight and temperature were measured at a predetermined time point. Spleens were collected from each group and weighed 7 days after different treatments. To evaluate systemic cytokine levels, another in vivo experiment was performed (*n* = 3). Serum samples were collected from mice at predetermined time points after different treatments and diluted for analysis using ELISA kits for IL-6 and TNF-α.

### Analysis of LIA-mediated in vivo DC maturation and immune responses upon irradiation

BALB/c mice (6–8 weeks) were subcutaneously transplanted with 1 × 10^6^ 4T1 tumour cells on the flank. The mice were treated with different formulations and irradiated for 30 min with or without a 665 nm laser at an intensity of 0.15 W/cm^2^. The treatments were performed three times every other day. Lymph nodes were surgically isolated 72 h after different treatments. The samples were then mechanically disrupted and digested in a solution containing 0.5 mg/mL collagenase IV at 37 °C for 40 min. Suspensions were filtered and washed with PBS containing 2% FBS. Single-cell suspensions were then stained with anti-CD11c-PE, anti-CD80-FITC and CD86-APC antibodies for 30 min. Flow cytometry analysis was performed. Spleens were surgically isolated 7 days after the final treatment. The samples were mechanically disrupted and incubated with ammonium chloride buffer to lyse erythrocytes and incubated with anti-CD3-PE and anti-CD8-APC before flow cytometry (BD Accuri C6 1.0.264.21, BD, USA).

### In vivo antitumour studies

To establish a bilateral tumour model, 1 × 10^6^ 4T1 tumour cells or CT26 tumour cells in PBS were subcutaneously transplanted in the left flank of BALB/c mice as the primary tumour. 6 days later, to form the second tumour (abscopal tumour), 4T1 tumour cells or CT26 tumour cells (1 × 10^6^) were subcutaneously injected in the right flank. The bilateral 4T1 or CT26 tumour-bearing mice were treated with different formulations with an equal dose of Ce6 (3 mg/kg). 4 h post injection, the primary tumours were irradiated for 30 min with a 665 nm laser at an intensity of 0.15 W/cm^2^. The treatments were performed three times every other day. The body weights of the mice and the volumes of the primary and abscopal tumours were monitored every other day. The tumour volumes were calculated as *V* = length × width^2^/2. When the length or width of the tumours was larger than 20 mm, or when the bodyweight loss reached 20%, the mice were euthanized^47^. To construct the lung metastasis model, 1 × 10^6^ 4T1 tumour cells were transplanted in the flank of the mice. The mice were intravenously injected with 4T1-luc cells (2 × 10^5^). Then the mice were i. v. injected with PBS, free Ce6 (3 mg/kg Ce6, with irradiation), DP-Ce6 (3 mg/kg Ce6, with irradiation) LIA (3 mg/kg Ce6, with or without irradiation). 4 h post-injection, the primary tumours were irradiated for 30 min (665 nm, 0.15 W/cm^2^). The treatments were performed three times every other day. 25 days after the different treatments, D-luciferin potassium salt at a concentration of 150 mg/kg body weight was administered via intraperitoneal injection. 10 min later, the mice were killed, and their lungs were excised. Fluorescence images were collected by a IVIS imaging system (with IVIS Spectrum software 4.5.5). Lung metastasis nodules were then manually counted, and lung tissues sections were subjected to H&E staining.

### Tumour cell re-challenge studies

BALB/c mice (6–8 weeks) were subcutaneously transplanted with 1 × 10^6^ 4T1 tumour cells or CT26 tumour cells on the left flank. When the tumour reached 80 mm^3^, the mice were treated with surgery or intravenously treated with LIA (3 mg/kg Ce6) and irradiated for 30 min with a 665 nm laser at an intensity of 0.15 W/cm^2^. The treatments were performed three times every other day. The mice were challenged on day 30 by subcutaneous transplanted of 1 × 10^6^ 4T1 tumour cells or CT26 tumour cells. Tumour growth was evaluated. The memory T cells were analysed by flow cytometry on day 30 before being challenged with tumour cells.

### The mechanism study of the immune responses

Tissue sections and flow cytometry experiments were performed to study the infiltration of cytotoxic T-lymphocytes into tumour tissue. For the tissue sections, the tumours were harvested from the different groups and fixed. Tumour slices were stained with antibodies for anti-CD4-Cy3 and ant-CD8-FITC. Nuclei were stained with DAPI. Slices were imaged with a fluorescence microscope. For flow cytometry experiments^48^, tumours were isolated and mechanically disrupted into small pieces. The samples were digested by 0.5 mg/mL collagenase IV at 37 °C for 40 min. Suspensions were filtered and washed. Cell suspensions were incubated with anti-CD3-PE, anti-CD8-APC and anti-CD4-FITC antibodies and then analysed by flow cytometry. Freshly isolated spleens were mechanically disrupted and incubated with ammonium chloride buffer to lyse erythrocytes and incubated with anti-CD3-PE, anti-CD8-APC and anti-CD4-FITC antibodies. For flow cytometry analysis of IFN-γ and IL-17 secreting T cells, the cells were first incubated with cell activation cocktail buffer (containing PMA, ionomycin and Brefeldin A) in a CO_2_ incubator at 37 °C for 6 h and then incubated with anti-CD8-APC or anti-CD4-PE antibody for surface staining. Cells were then fixed, permeabilized and stained for intracellular IFN-γ and IL-17 antibodies for flow cytometry. For analysis of memory T cells, cell suspension of spleens was prepared as mentioned above. Then the cell suspensions were stained with anti-CD8-APC, anti-CD44-FITC and anti-CD62L-Percp Cy5.5 for flow cytometry analysis (BD Accuri C6 1.0.264.21, BD, USA).

### Statistical analysis

All values are expressed as mean ± standard deviation (s.d.). Statistical analyses were calculated using GraphPad Prism 7. Statistically significant differences between the two groups were determined using a two-tailed Student’s *t*-test. For multiple comparisons, one-way ANOVA was used.

### Reporting summary

Further information on research design is available in the Nature Research Reporting Summary linked to this article.

## Supplementary information

Supplementary Information

Reporting Summary

## Data Availability

Transcriptomics sequencing data are available from the Sequence Read Archive under accession code PRJNA747617. The remaining data are available within the Article, Supplementary Information or Source Data file. Source data are provided with this paper.

## Source data

Source Data
