# Supplementary material for: An amphiphilic dendrimer as a light-activable immunological adjuvant for in situ cancer vaccination
Source: Nat Commun. 2021 Aug 16;12:4964. doi: 10.1038/s41467-021-25197-z (PMC8368031; doi:10.1038/s41467-021-25197-z)
Supplement: Supplementary file 1 — Supplementary Information [file 41467_2021_25197_MOESM1_ESM.pdf]

Supporting information

An amphiphilic dendrimer as a light-activable immunological adjuvant for in situ cancer vaccination

Yongchao Wang[a,b,c,†], Ningqiang Gong[b,c,†,*], Chi Ma[a], Yuxuan Zhang[b,c], Hong Tan[b,c,d,], Guangchao Qing[b,c], Jimei Zhang[b,c], Yufei Wang[b,c], Jinjin Wang[b,c], Shizhu Chen[b,c], Xianlei Li[b,c], Qiankun Ni[b,c], Yuan Yuan[b,c], Yaling Gan[b,c], Junge Chen[b,c], Fangzhou Li[b,c], Jinchao Zhang[e], Caiwen Ou[f], Yongxiang Zhao[d,*], Xiaoxuan Liu[a,*] and Xing-Jie Liang[b,c,*]

[a]State Key Laboratory of Natural Medicines and Jiangsu Key Laboratory of Drug Discovery for Metabolic Diseases, Center of Drug Discovery, Center of Advanced Pharmaceutics and Biomaterials, China Pharmaceutical University, 24 Tong Jia Xiang, Nanjing, 210009, China.

[b]Laboratory of Controllable Nanopharmaceuticals, Chinese Academy of Sciences (CAS) Center for Excellence in Nanoscience and CAS Key Laboratory for Biomedical Effects of Nanomaterials and Nanosafety, National Center for Nanoscience and Technology, Beijing 100190, China.

[c]University of Chinese Academy of Sciences, Beijing 100049, China.

[d]National Center for International Biotargeting Theranostics, Guangxi Key Laboratory of Biotargeting Theranostics, Collaborative Innovation Center for Targeting Tumour Theranostics, Guangxi Medical University, Guangxi 530021, China.

[e]Key Laboratory of Medicinal Chemistry and Molecular Diagnosis of the Ministry of Education, College of Chemistry & Environmental Science, Hebei University, Baoding 071002, China.

[f]Dongguan Hospital of Southern Medical University, Southern Medical University, Guangdong Provincial Key Laboratory of Shock and Microcirculation，Guangzhou 510280, China.

*Email: gongnq@nanoctr.cn; yongxiang_zhao@126.com: xiaoxuanliucpu@163.com; liangxj@nanoctr.cn

[†]These authors contributed equally to this work.

Table of contents

Supplementary Figure 1. Synthetic route of non-responsive amphiphilic dendrimer（N-HAD） 4
Supplementary Figure 2. ^{1}H-NMR spectra of the intermediate products of HAD........5
Supplementary Figure 3. ^{1}H-NMR spectra of the intermediate products of N-HAD....6
Supplementary Figure 4. Characterization of HAD NPs..7
Supplementary Figure 5. Stability of LIA NPs in PBS and fetal bovine serum (FBS) containing medium 8
Supplementary Figure 6. Characterization of the hypoxia microenvironment induced by LIA under NIR laser irradiation 9
Supplementary Figure 7. ESI-MS spectra of residues after irradiation. 9
Supplementary Figure 8. Cumulative release of Ce6 from LIA NPs.........................10
Supplementary Figure 9. ESI-MS spectrum of rHAD reduced by sodium dithionite .10
Supplementary Figure 10. Endotoxin levels and biocompatibilities of different molecules/nanoparticles 11
Supplementary Figure 11. In vitro cytotoxicity of tumour cells treated with free Ce6 or LIA at different concentrations.. 11
Supplementary Figure 12. Cellular uptake of free Ce6 and LIA 12
Supplementary Figure 13. Hypoxia and ROS generation inside tumour cells induced by LIA upon irradiation...13
Supplementary Figure 14. Adjuvant effect of rHAD combine with OVA 14
Supplementary Figure 15. Transcriptomics analysis of BMDCs treated with different formulations 15
Supplementary Figure 16. rHAD enhances the maturation of BMDCs by activating TLR7 signaling pathway. 16
Supplementary Figure 17. Neoantigens release from LIA+L-treated tumour cells and the generation of antigen-specific immune responses. 18
Supplementary Figure 18. Blood compatibility analysis after incubation with HAD NPs 19
Supplementary Figure 19. The stability of LIA NPs in vivo 20
Supplementary Figure 20. LIA induced reduced systemic toxicity 21
Supplementary Figure 21. Tumour inhibition in a 4T1 bilateral tumour model..........23
Supplementary Figure 22. Tumour inhibition in a CT26 bilateral tumour model 24
Supplementary Figure 23. Flow cytometry gating strategy for flow cytometry analysis 26
Supplementary Figure 24. Images of H&E staining of major organs of 4T1 bilateral tumour model...27
Supplementary Figure 25. Images of H&E staining of major organs of CT26 bilateral

tumour model..28
Supplementary Figure 26. Biodistribution, tumour accumulation and antitumour efficiency of different nanoparticles. 30
Supplementary Figure 27. Analysis of memory T cells in $CD4^{+}$ T cells and flow cytometry gating strategy for the analysis of memory T cells 31
Supplementary Table 1. Dynamic molecular docking of HAD and rHAD head groups with TLR7...32
Supplementary Table 2. Characterizations of the prepared nanoparticles. 32

Supplementary Figurers

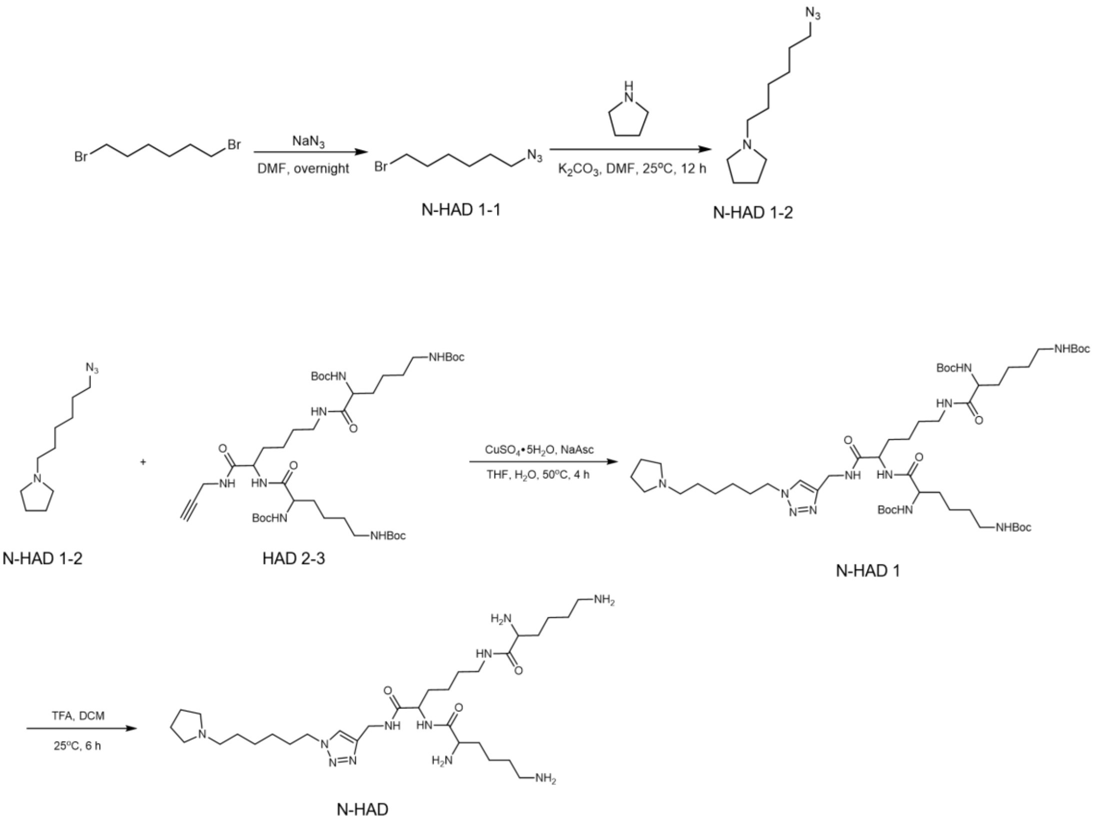

Supplementary Figure 1. Synthetic route of non-responsive amphiphilic dendrimer（N-HAD）.

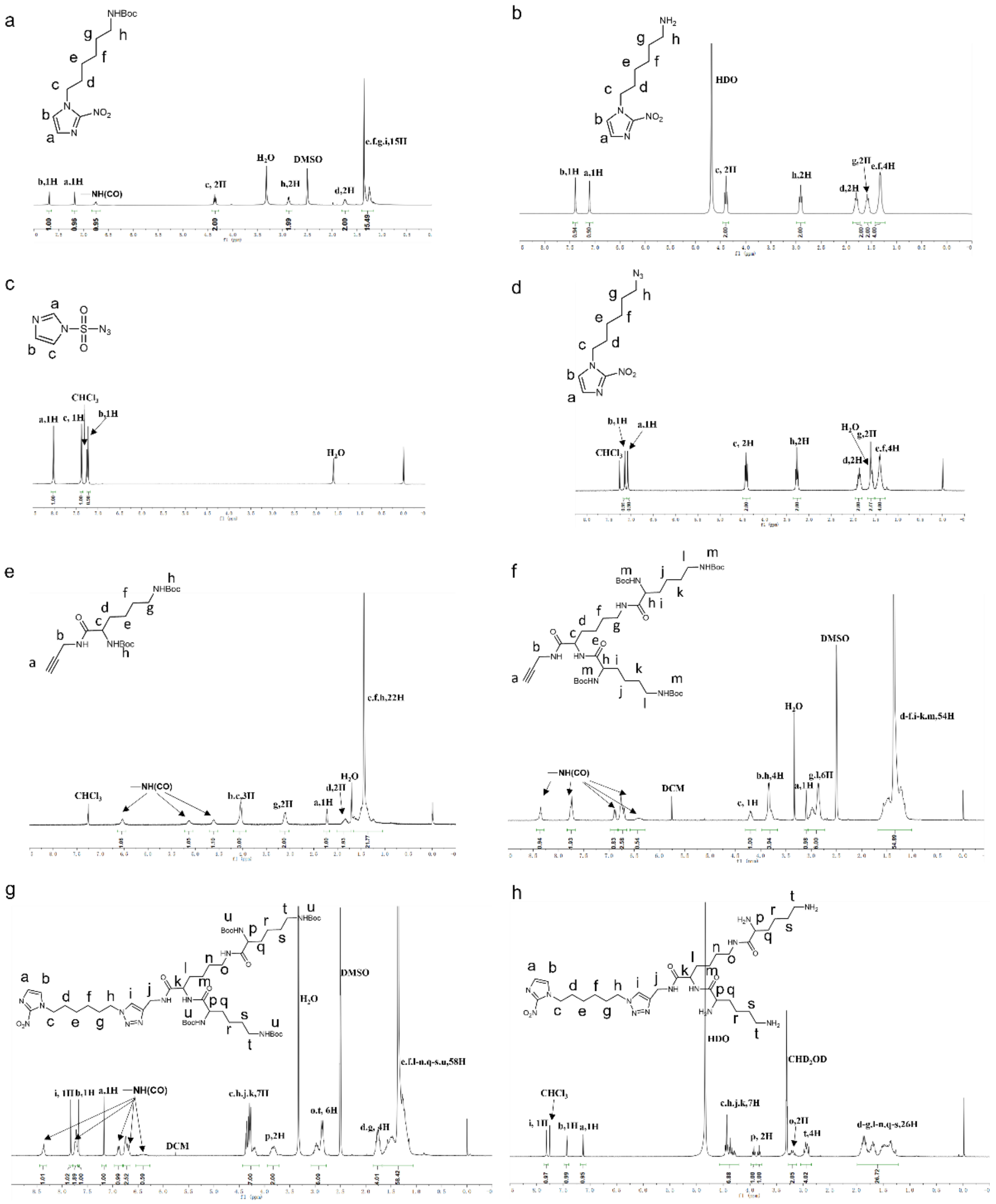

Supplementary Figure 2. ^{1}H-NMR spectra of the intermediate products of HAD. **a**, ^{1}H-NMR spectrum of HAD 1-1 in $CDCl_3$. **b**, ^{1}H-NMR spectrum of HAD 1-2 in D_2O. **c**, ^{1}H-NMR spectrum of imidazole-1-sulfonyl azide in $CDCl_3$. **d**, ^{1}H-NMR spectrum of HAD 1-3 in $CDCl_3$. **e**, ^{1}H-NMR spectrum of HAD 2-1 in $CDCl_3$. **f**, ^{1}H-NMR spectrum of HAD 2-3 in DMSO-d_6. **g**, ^{1}H-NMR spectrum of HAD 1 in DMSO-d_6. **h**, ^{1}H-NMR spectrum of HAD in MeOD/$CDCl_3$ = 3/1.

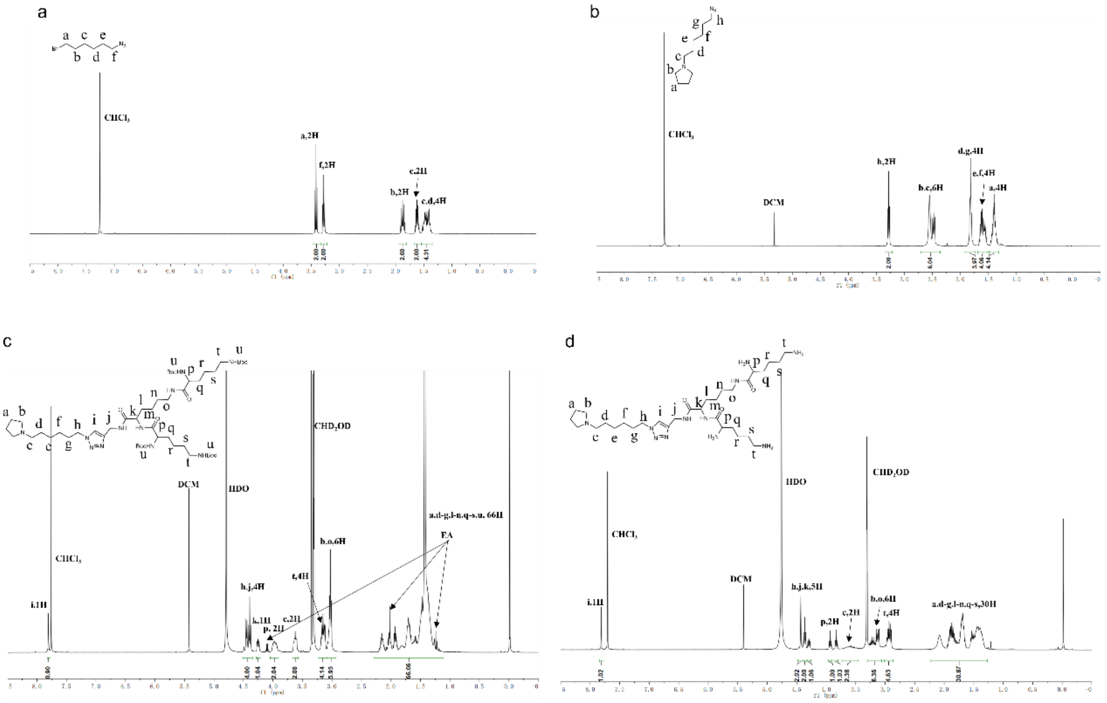

Supplementary Figure 3. ^{1}H-NMR spectra of the intermediate products of N-HAD. **a**, ^{1}H-NMR spectrum of N-HAD 1-1 in $CDCl_3$. **b**, ^{1}H-NMR spectrum of N-HAD 1-2 in $CDCl_3$. **c**, ^{1}H-NMR spectrum of N-HAD1 in MeOD/$CDCl_3$ = 3/1. **d**, ^{1}H-NMR spectrum of N-HAD in MeOD/$CDCl_3$ = 3/1.

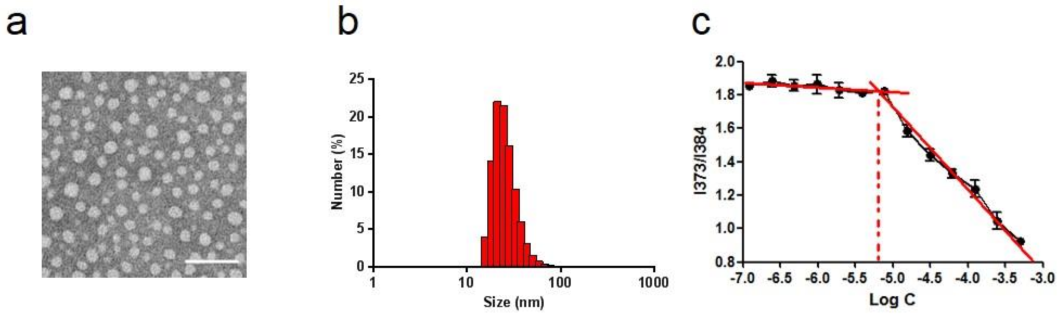

Supplementary Figure 4. Characterization of HAD NPs. **a**, TEM image of HAD NPs (LIA without Ce6 loading). Scale bar: 100 nm. Experiments were performed three times independently, one representative image is shown. **b**, DLS size distribution of HAD. Experiments were performed three times independently, one representative data is shown. **c**, the critical micelle concentration of HAD measured using the fluorescent dye pyrene. Data in **c** are presented as mean ± s.d. from three independent experiments (n = 3).

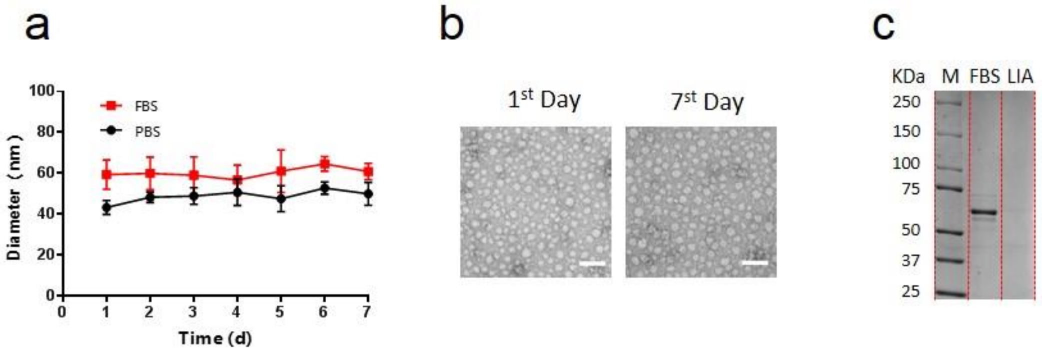

Supplementary Figure 5. Stability of LIA NPs in PBS and fetal bovine serum (FBS) containing medium. **a**, Diameter changes of LIA NPs incubated in PBS and FBS, data are shown as mean ± s.d. (n= 3). **b**, TEM images of the LIA NPs dispersed in FBS containing medium. Scale bar: 100 nm. **c**, SDS-PAGE analysis of protein adsorption of LIA NPs. 10 mg LIA NPs were incubated with 10% FBS-containing medium for 2 h and then the LIA nanoparticles were collected by centrifugation. After washed with PBS for 3 times, LIA was loaded on a gel and the protein absorption was measured, 10 ug FBS was used as a control. M, protein ladder. Experiments in **b**,**c** were performed three times independently, representative images are shown.

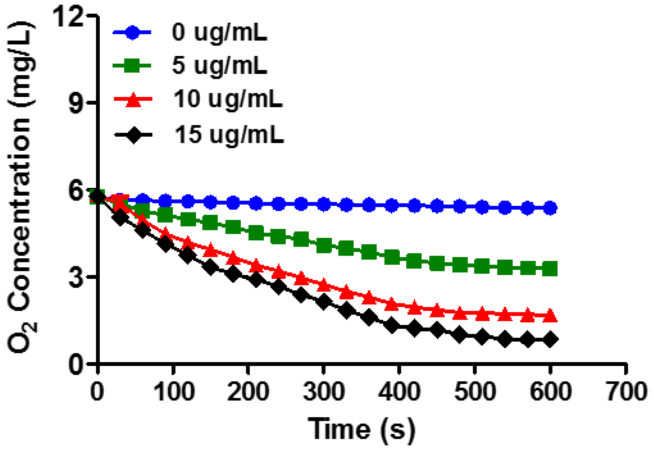

Supplementary Figure 6. Characterization of the hypoxia microenvironment induced by LIA under NIR laser irradiation. O_2 concentration in medium containing different concentrations of LIA (irradiated with 665 nm laser (0.15 W/cm^2) was measured using a dissolved oxygen meter. Data are presented as mean ± s.d. (n = 3).

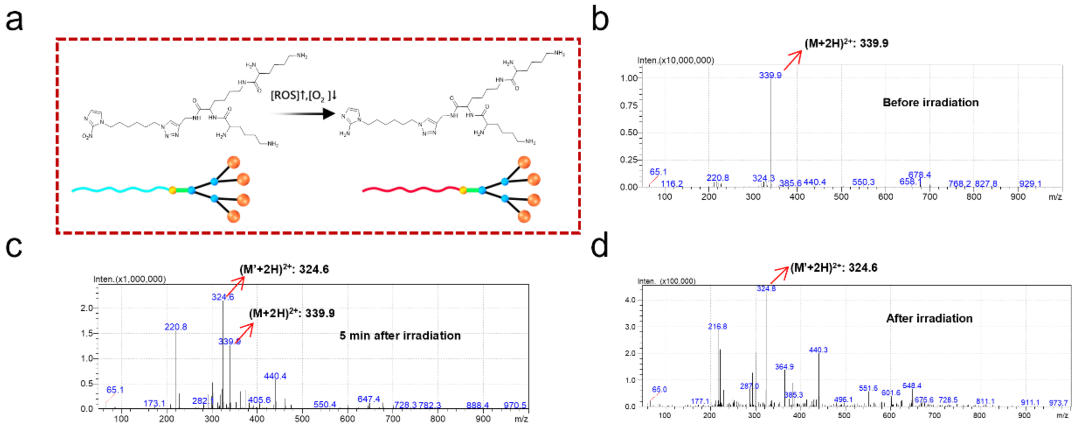

Supplementary Figure 7. ESI-MS spectra of residues after irradiation. **a**, schematic illustration of the group change of HAD before and after irradiation. **b**-**d**, ESI-TOF mass spectrum of HAD(**b**), LIA NPs 5 min, (**c**) and 24 h (**d**) after irradiation.

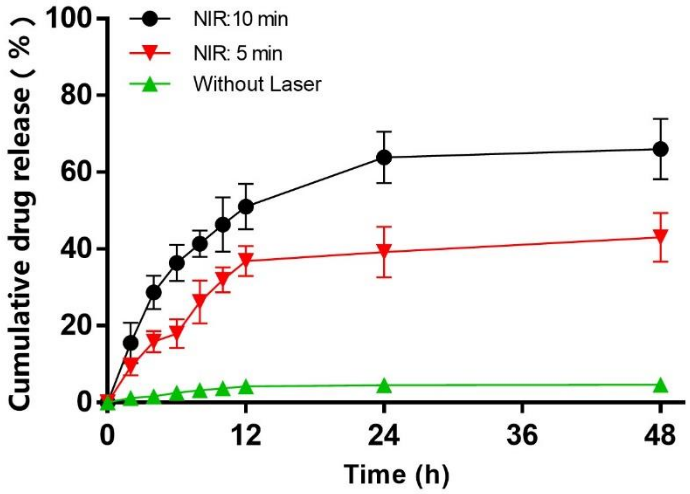

Supplementary Figure 8. Cumulative release of Ce6 from LIA NPs. LIA NPs were loaded in a dialysis tube and the dialysis tube was immersed in a PBS buffer in the presence or absence of NIR light irradiation for 5 or 10 min. Cumulative Ce6 release was evaluated by measuring the absorbance of Ce6. Data are shown as mean ± s.d. (n = 4)

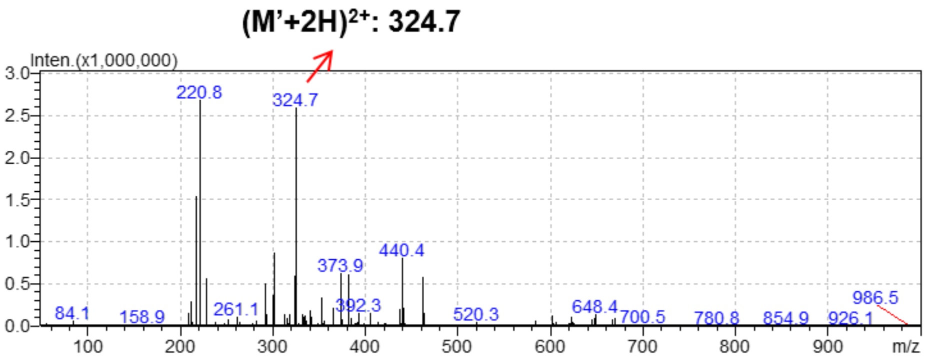

Supplementary Figure 9. ESI-MS spectrum of rHAD reduced by sodium dithionite.

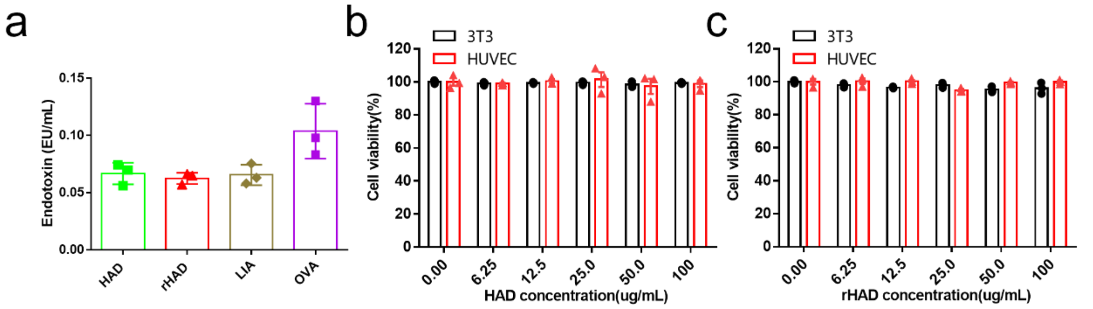

Supplementary Figure 10. Endotoxin levels and biocompatibilities of different molecules/nanoparticles. **a**, Endotoxin levels of the formulations were detected using a chromogenic LAL endotoxin assay kit. **b**,**c**, Cell viabilities of NIH3T3 cells and HUVEC cells were evaluated after the cells were incubated with HAD (**b**) and rHAD (**c**) for 24 h. Data were shown as mean ± s.d. (n = 3).

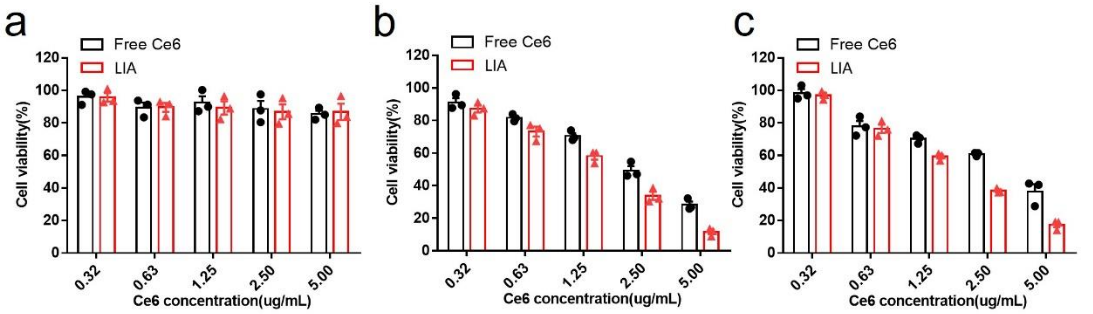

Supplementary Figure 11. In vitro cytotoxicity of tumour cells treated with free Ce6 or LIA at different concentrations. **a**, 4T1 cells were incubated with free Ce6 or LIA at different concentrations in the absence of NIR light irradiation. b,c, 4T1 cells or CT26 cells were incubated with free Ce6 or LIA and with NIR light irradiation (665 nm, 0.15 W/cm^2, for 2 min). Cell viabilities of 4T1 cells (**b**) and CT26 cells (**c**) were measured after additional12 h incubation. Data were shown as mean ± s.d. (n = 3).

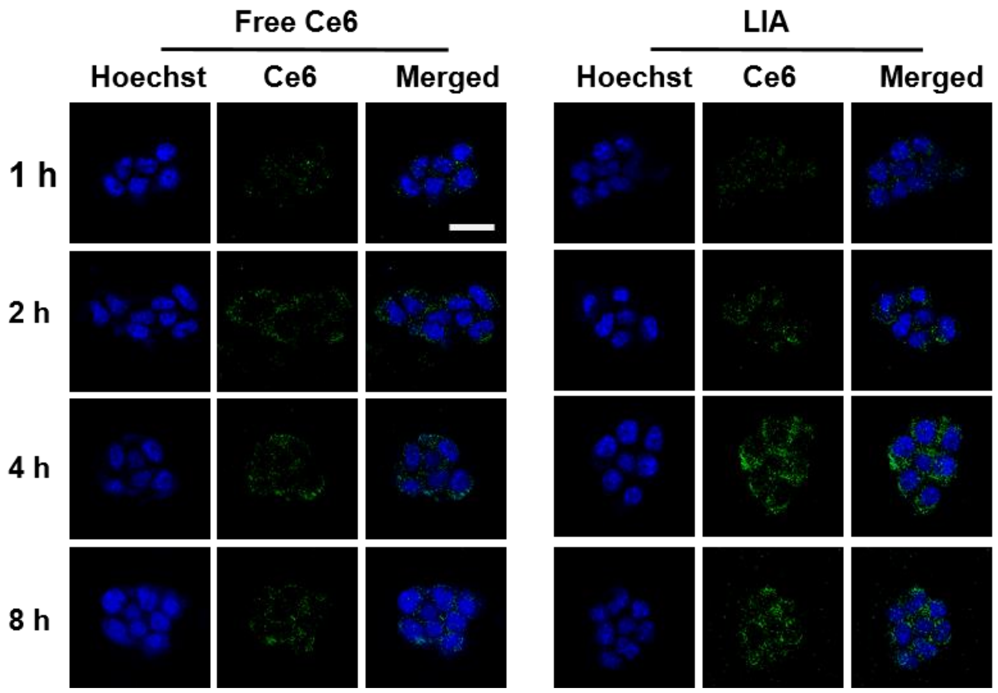

Supplementary Figure 12. Cellular uptake of free Ce6 and LIA. 4T1 cells were treated with free Ce6 or LIA at different time points with equivalent Ce6 dose 5 μg/mL. Blue and green represent Hoechst and Ce6 fluorescence, respectively. Scale bar: 20 μm. Experiments were performed three times independently, representative images are shown.

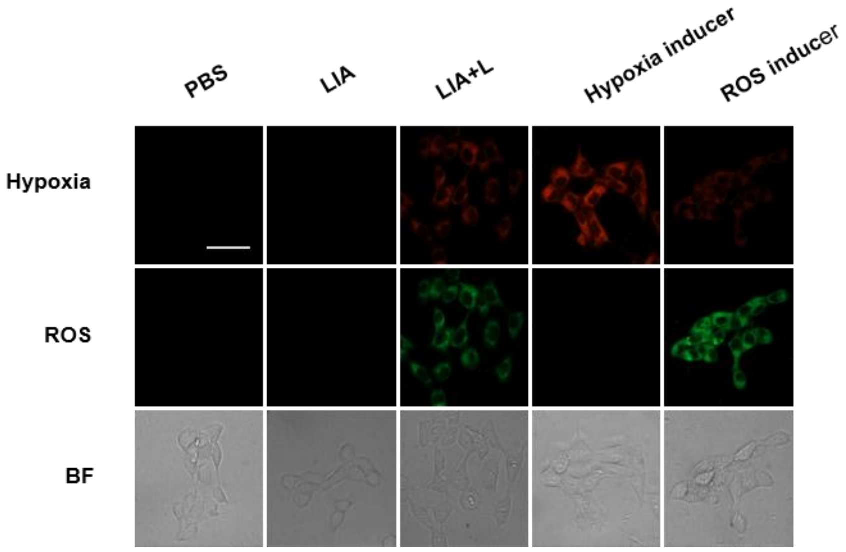

Supplementary Figure 13. Hypoxia and ROS generation inside tumour cells induced by LIA upon irradiation. Confocal images of 4T1 tumour cells stained with ROS/hypoxia probes after different treatments: PBS, LIA without irradiation, LIA with irradiation, and positive control groups. Scale bar: 50 μm. Experiments were performed three times independently, representative images are shown.

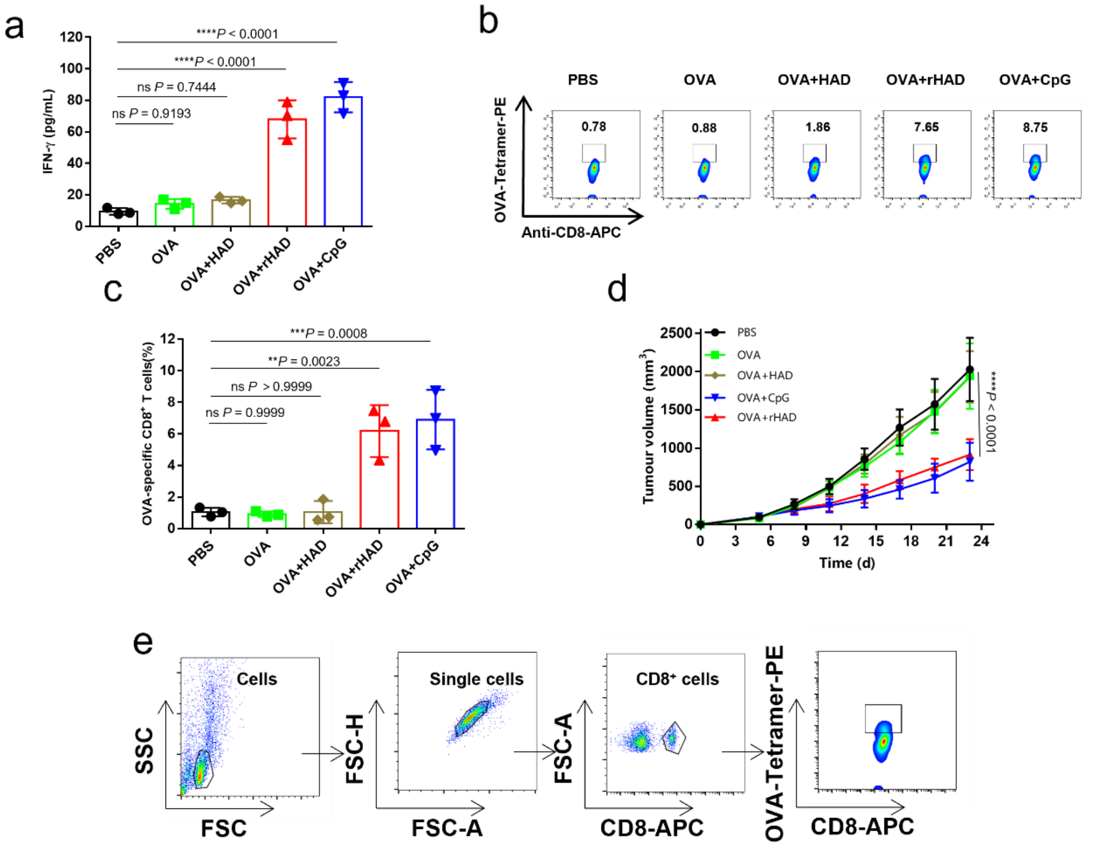

Supplementary Figure 14. Adjuvant effect of rHAD combine with OVA. **a**, IFN-γ secretion by OT-I CD8+ T cells incubated with BMDCs treated with OVA alone or OVA plus different formulations. **b**, Representative flow dot plots of OVA tetramer+ CD8+ T cells in spleen of C57BL/6 mice immunized with different formulations. **c**, Percentage of OVA tetramer+ CD8+ T cells measured by flow cytometry. **d**, B16-OVA tumour-bearing C57BL/6 mice (n = 5) were treated on days 5 and 12 with OVA+rHAD or the control formulations (PBS, OVA, OVA+HAD and OVA+CpG) Tumour volumes were measured every three days. **e**, Flow cytometry gating strategy for the analysis of OVA tetramer+ CD8+ T cells. Data in (**a**,**c**) are presented as mean ± s.d. (n = 3), data in **d** are presented as mean ± s.d. (n = 5). Statistically significant differences between groups were identified by one-way ANOVA. ****$P < 0.0001$, ***$P < 0.001$, **$P < 0.01$, *$P < 0.05$.

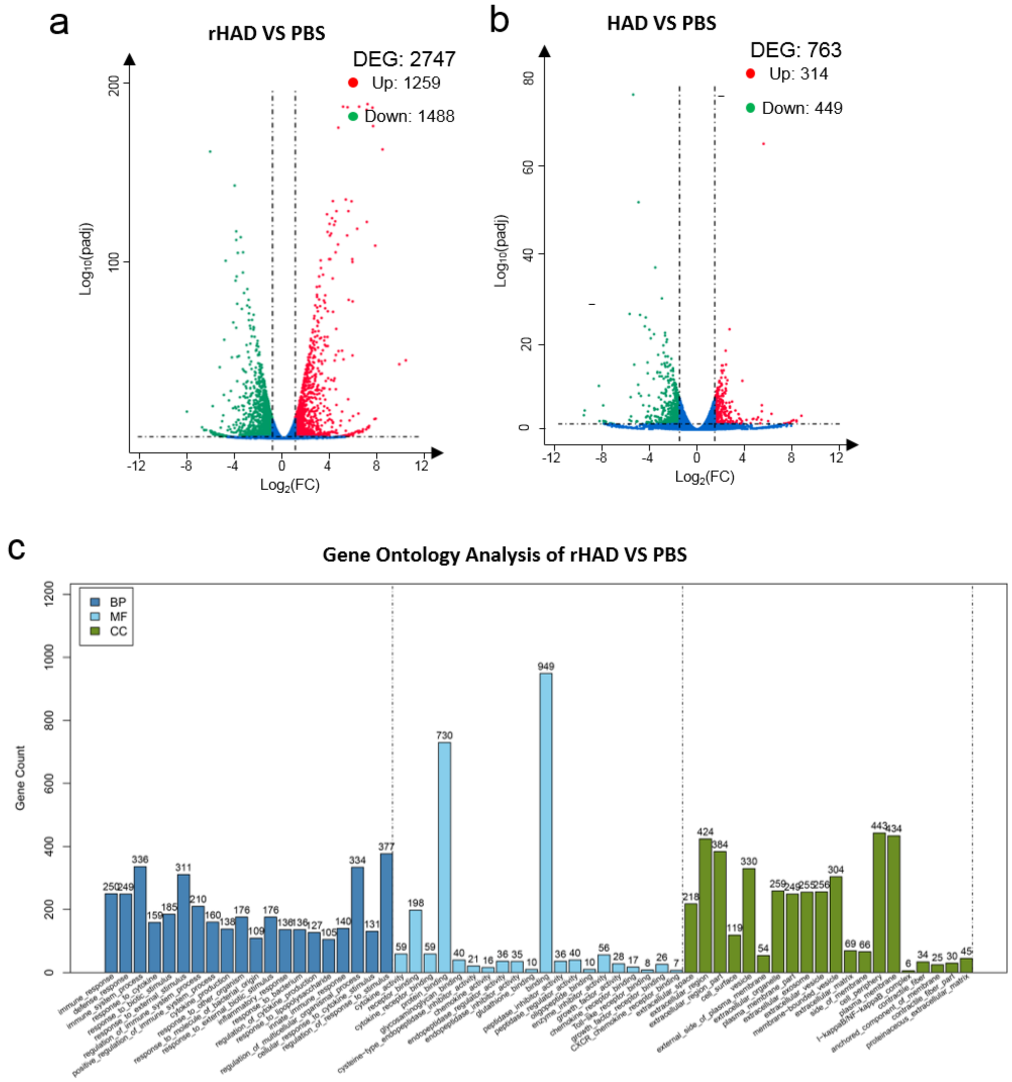

Supplementary Figure 15. Transcriptomics analysis of BMDCs treated with different formulations. **a**, Volcano plots of DEGs with rHAD treatment compared with PBS treatment. **b**, Volcano plots of DEGs with HAD treatment compared with PBS treatment. **c**, GO enrichment analysis of differentially expressed genes between rHAD and PBS treated BMDCs.

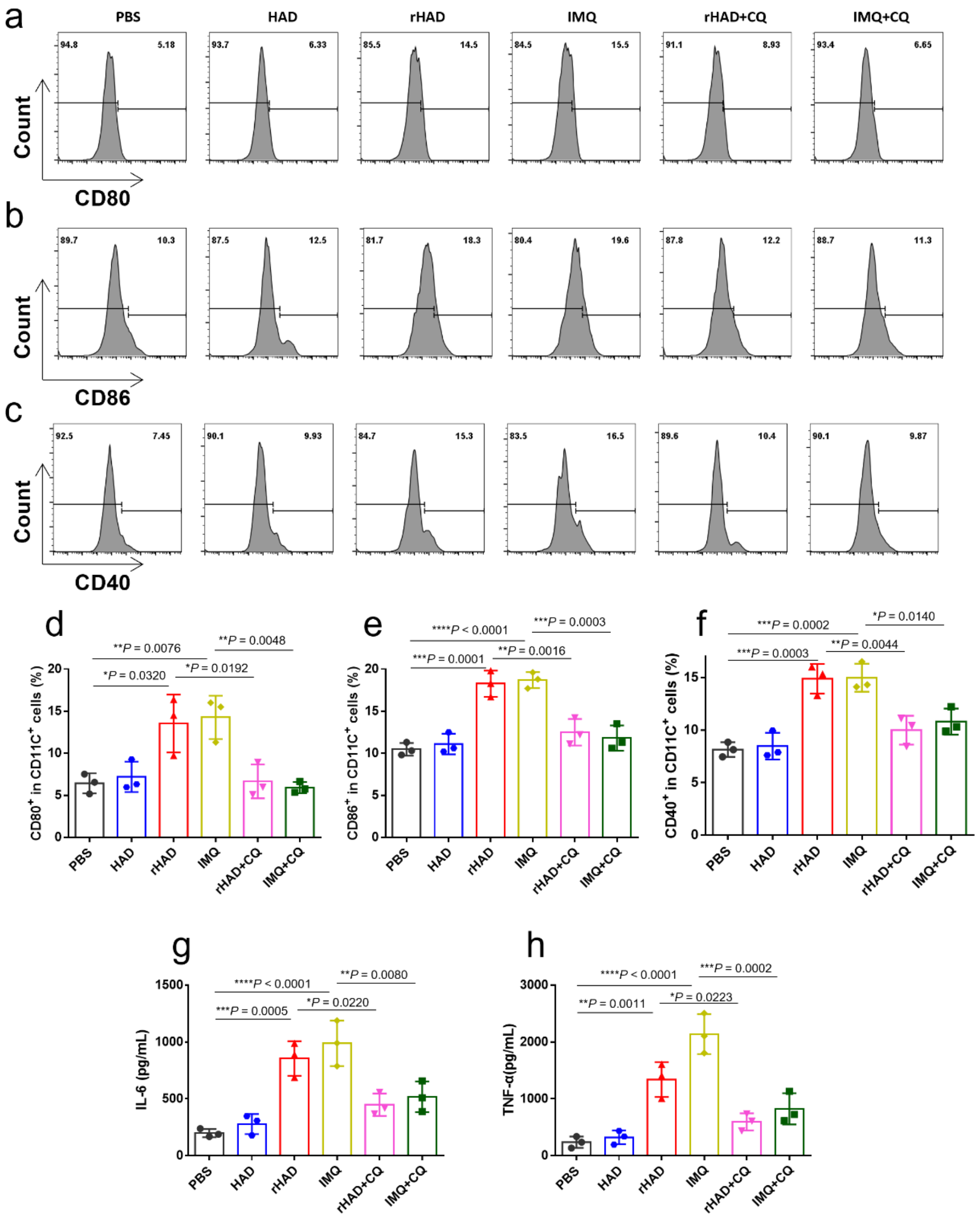

Supplementary Figure 16. rHAD enhances the maturation of BMDCs by activating TLR7 signaling pathway. Flow cytometry analysis of bone marrow-derived dendritic cells (BMDCs) maturation after the cells were incubated with PBS, rHAD (10 ug/mL), HAD (10 ug/mL), IMQ (imiquimod, 2ug/mL), rHAD + CQ (chloroquine 5 ug/mL, rHAD 10 ug/mL) or IMQ+CQ (chloroquine 5 ug/mL, imiquimod 2ug/mL) for 12 h. **a**-**c**, Flow cytometric histograms of CD80 (**a**), CD86 (**b**) and CD40 (**c**) expressions in BMDCs after different

treatments (gated on $CD11C^+$ cells). **d**-**f**, Quantification of CD80 (**d**), CD86 (**e**) and CD40 (**f**) expressions after BMDCs were treated with different formulations. **g**,**h**, TNF-α (**g**) and IL-6 (**h**) concentrations in the supernatants of BMDCs after 12 h incubation with different formulations (n = 3). Data in (**d**-**h**) are presented as mean ± s.d. (n = 3) and statistically significant differences between groups were identified by one-way ANOVA. ****$P < 0.0001$, ***$P < 0.001$, **$P < 0.01$, *$P < 0.05$.

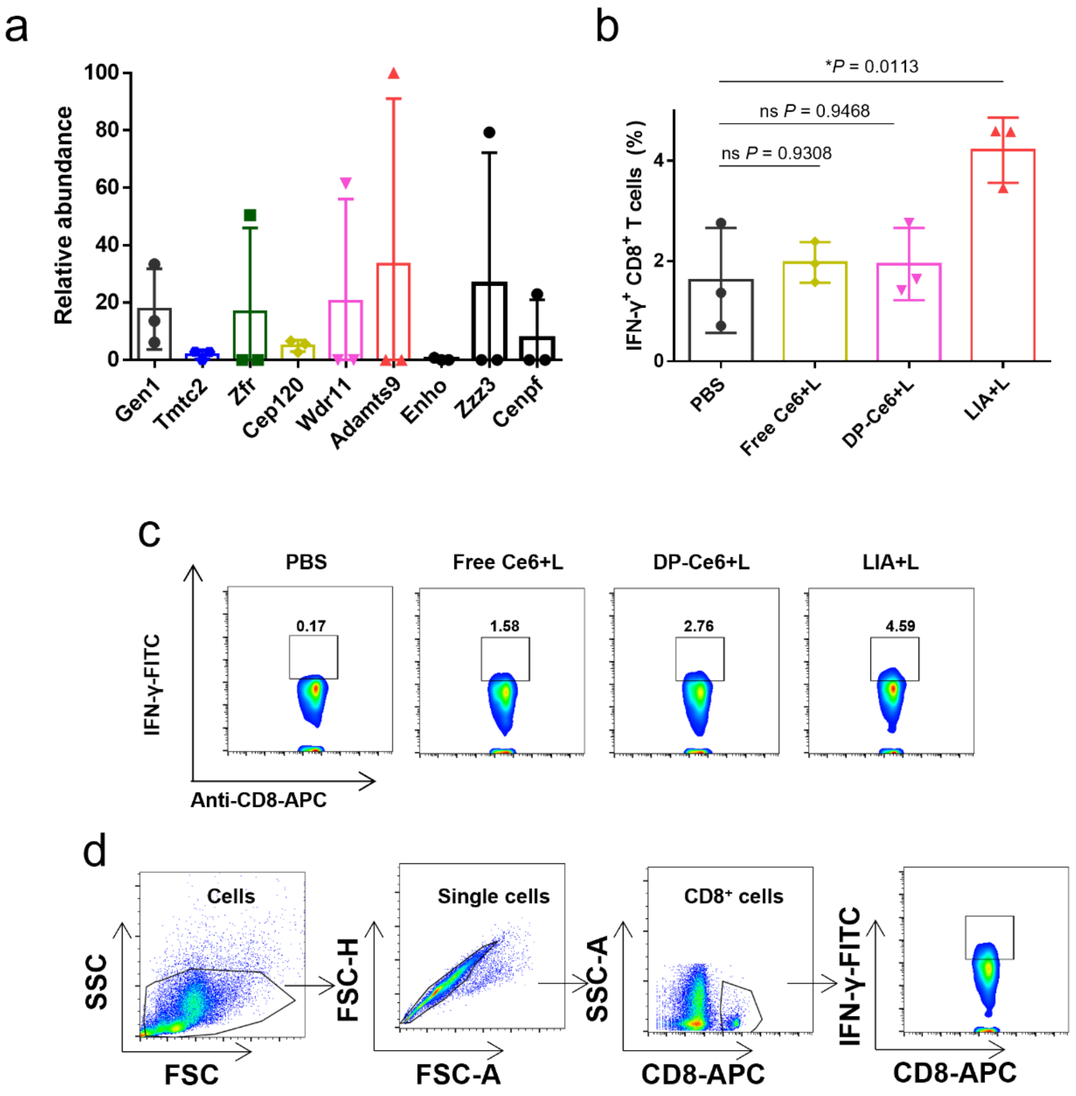

Supplementary Figure 17. Neoantigens release from LIA+L-treated tumour cells and the generation of antigen-specific immune responses. **a**, Relative abundance of neoantigen peptides determined in the supernatant of the 4T1 tumour cells treated with LIA after NIR light irradiation (665 nm, 0.15 W/cm^2) for 2 min. **b**, The frequency of IFN-γ$^+$ CD8$^+$ T cells in splenocytes isolated from mice with different treatments and stimulated ex vivo with supernatant of tumour cells treated with LIA+L. **c**, Representative flow cytometric analysis of IFN-γ$^+$ CD8$^+$ T cells in **b**. **d**, Flow cytometry gating strategy for the analysis of IFN-γ$^+$ CD8$^+$ T cells. Data in (**a**,**b**) are presented as mean ± s.d. (n = 3) and statistically

significant differences between groups were identified by one-way ANOVA. ****$P < 0.0001$, ***$P < 0.001$, **$P < 0.01$, *$P < 0.05$.

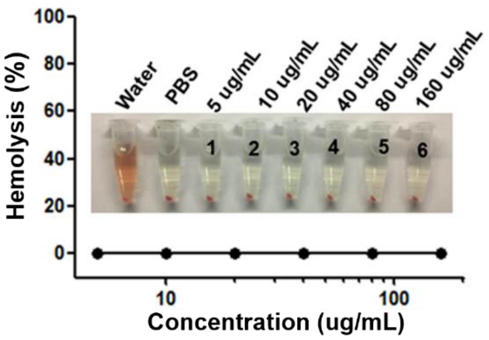

Supplementary Figure 18. Blood compatibility analysis after incubation with HAD NPs. The mouse red blood cells were used to incubate with HAD NPs. Water was used as a positive control and saline was used as a negative control. Hemolysis percentage was quantified by the releasing of hemoglobin into the buffer.

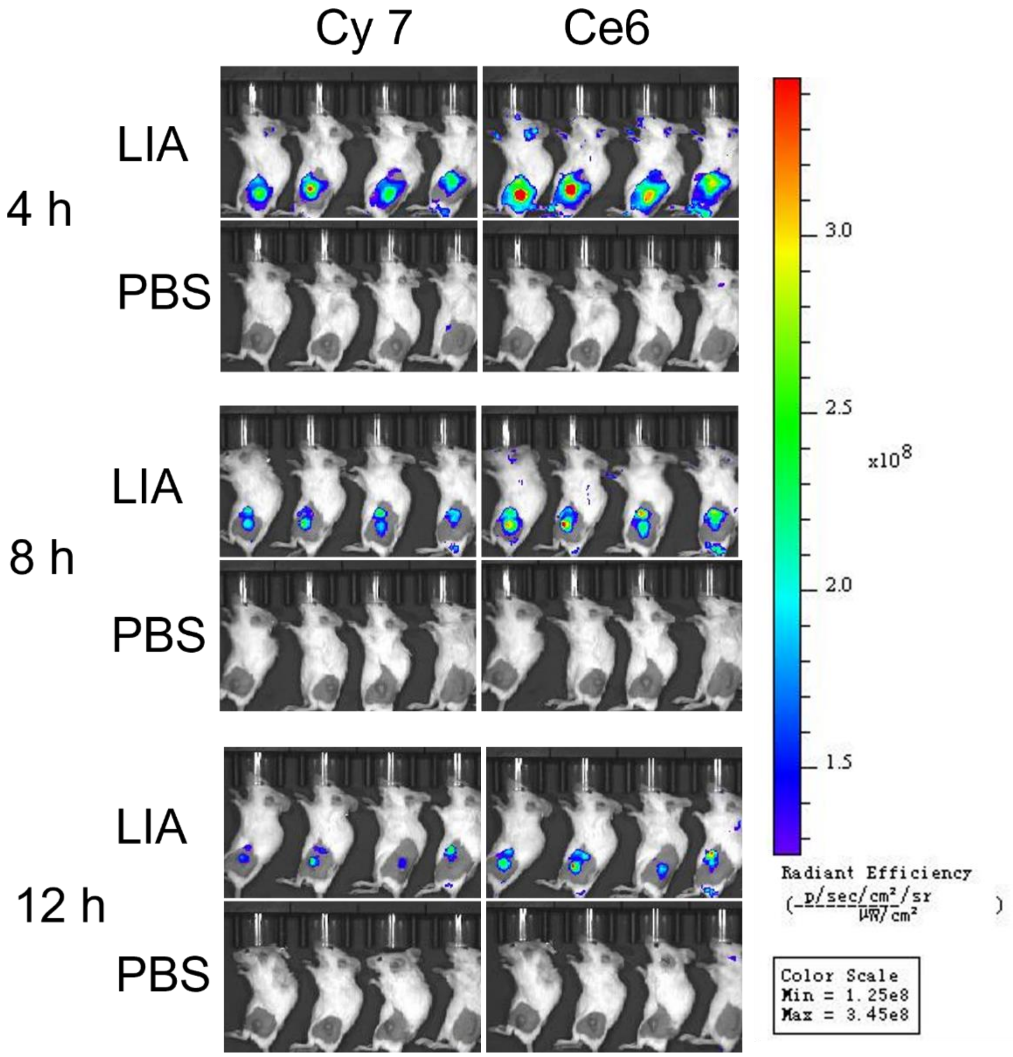

Supplementary Figure 19. The stability of LIA NPs in vivo. Fluorescence images of the 4T1 tumor-bearing mice at 4, 8 and 12 h post intravenous administration of NPs labeled with Cy7. The fluorescence signal of Cy7 and Ce6 was co-localized at the tumour site at 4, 8 and 12 h post intravenous administration, 4 mice per group.

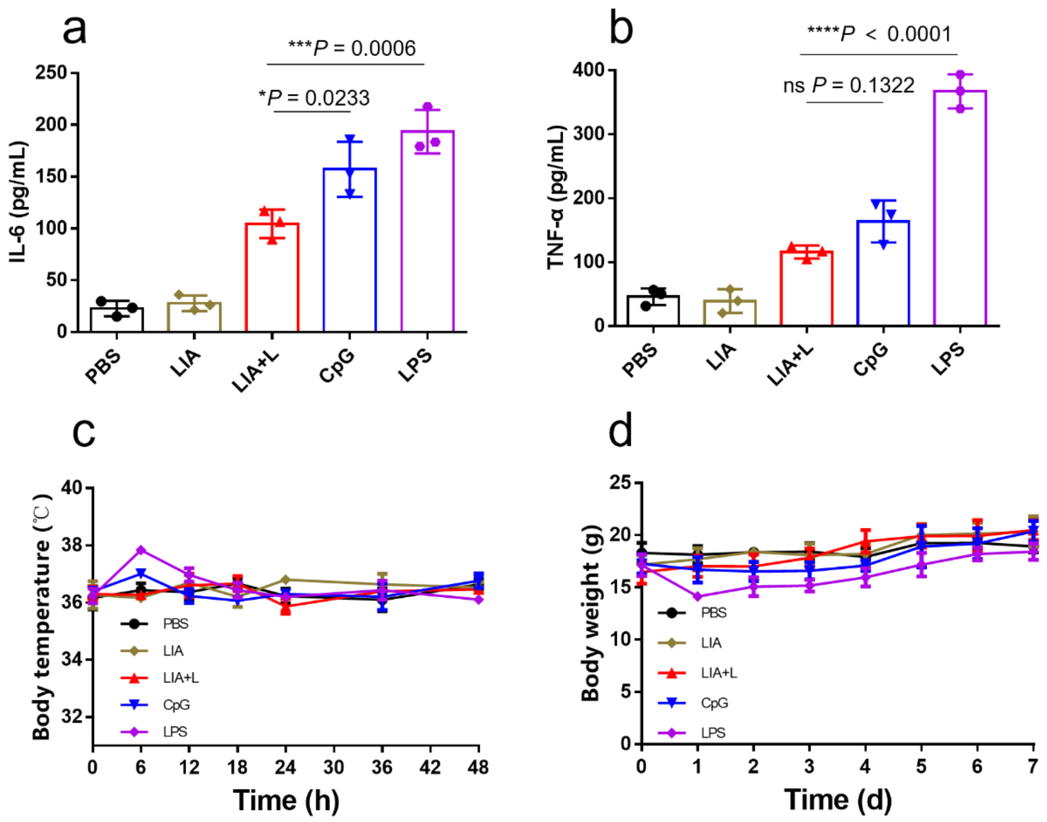

Supplementary Figure 20. LIA induced reduced systemic toxicity. **a**,**b**, ELISA analysis of TNF-α (**a**) and IL-6 (**b**) concentrations in serum collected from mice 2 h after different treatments (PBS, LIA, LIA+L, CpG and LPS). **c**,**d**, Body weight (**c**) and body temperature (**d**) of the 4T1 tumour-bearing BALB/c mice with different treatments over time. Data in (**a**-**d)** are presented as mean ± s.d. (n = 3) and statistically significant differences between groups were identified by one-way ANOVA. ****P < 0.0001, ***P < 0.001, **P < 0.01, *P < 0.05.

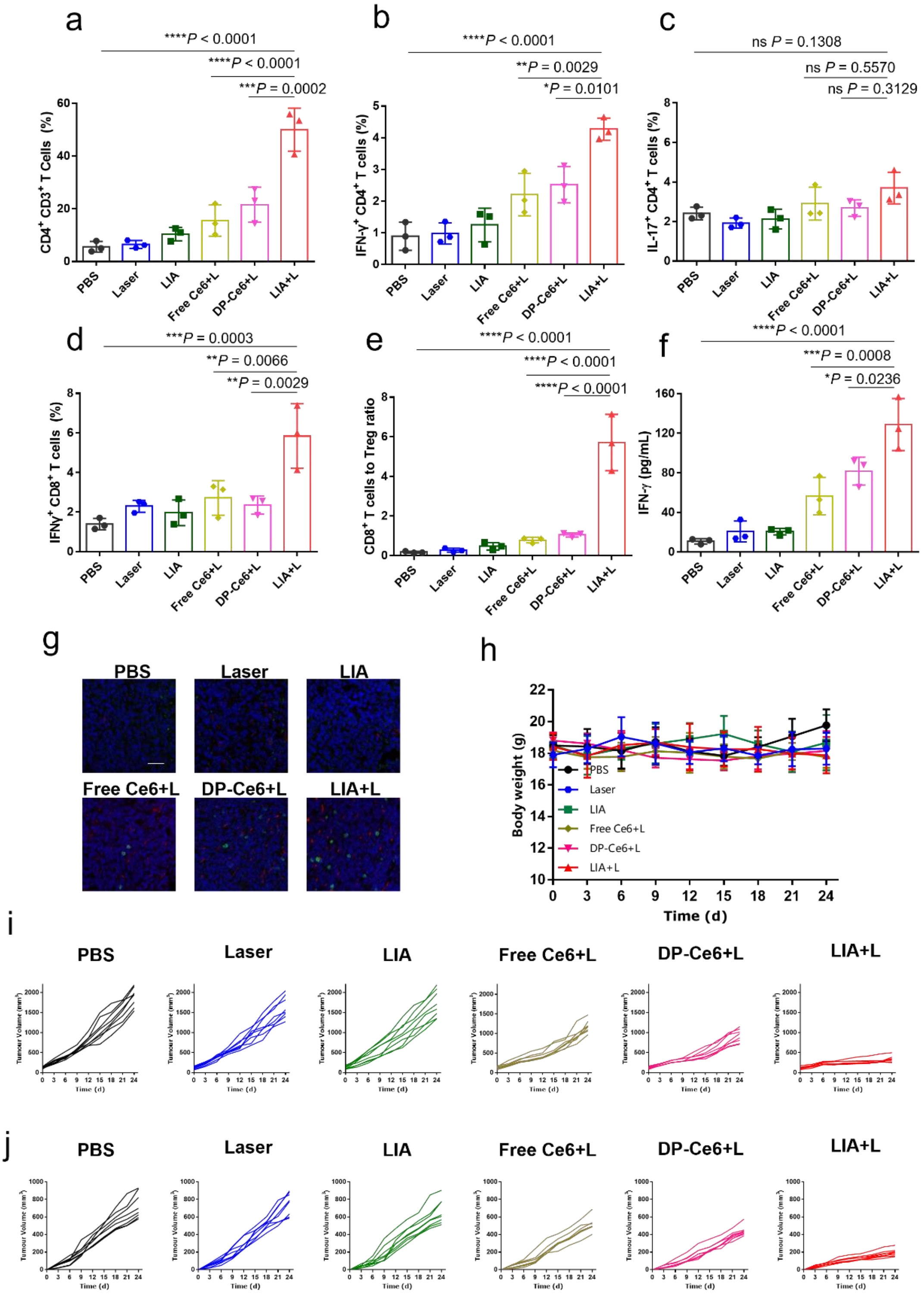

Supplementary Figure 21. Tumour inhibition in a 4T1 bilateral tumour model. Bilateral 4T1 tumour-bearing mice were treated with different formulations. 7 days after the final treatment, mice were euthanized, abscopal tumour tissue was collected and the immune cell infiltration was analyzed using flow cytometry. **a**, The percentage of

$CD4^+CD3^+$ T cell in abscopal tumour tissue. **b**, The percentage of IFN-γ secreting T cells in $CD4^+$ T cells. **c**, The percentage of IL-17 secreting T cells in $CD4^+$ T cells. **d**, The percentage of IFN-γ secreting T cells in $CD8^+$ T cells. **e**, Statistical analysis of $CD3^+$ $CD8^+$ T cells to $CD4^+$ $CD25^+$ $foxp3^+$T cell ratio in abscopal tumours tissues. **f**, The levels of IFN-γ in the abscobal tumour microenvironment. **g**, Representative immunofluorescence images of $CD8^+$ (green) and $CD4^+$ (red) infiltrated into abscopal tumours, scale bar: 50 μm. Experiments in **g** were performed three times independently, representative images are shown. **h**, Mice body weight was monitored during the anti-tumour therapy period (n=8). **i**,**j**, Individual tumour growth curves of 4T1 tumour model. Individual tumour growth curves of the primary tumours (**i**) and abscopal tumours (**j**) in 4T1 bilateral tumour model (n=8) treated with various formulations are shown. Data in (**a**-**f**) are presented as mean ± s.d. (n = 3), data in **h** are presented as mean ± s.d. (n = 8) and statistically significant differences between groups were identified by one-way ANOVA. ****$P < 0.0001$, ***$P < 0.001$, **$P < 0.01$, *$P < 0.05$.

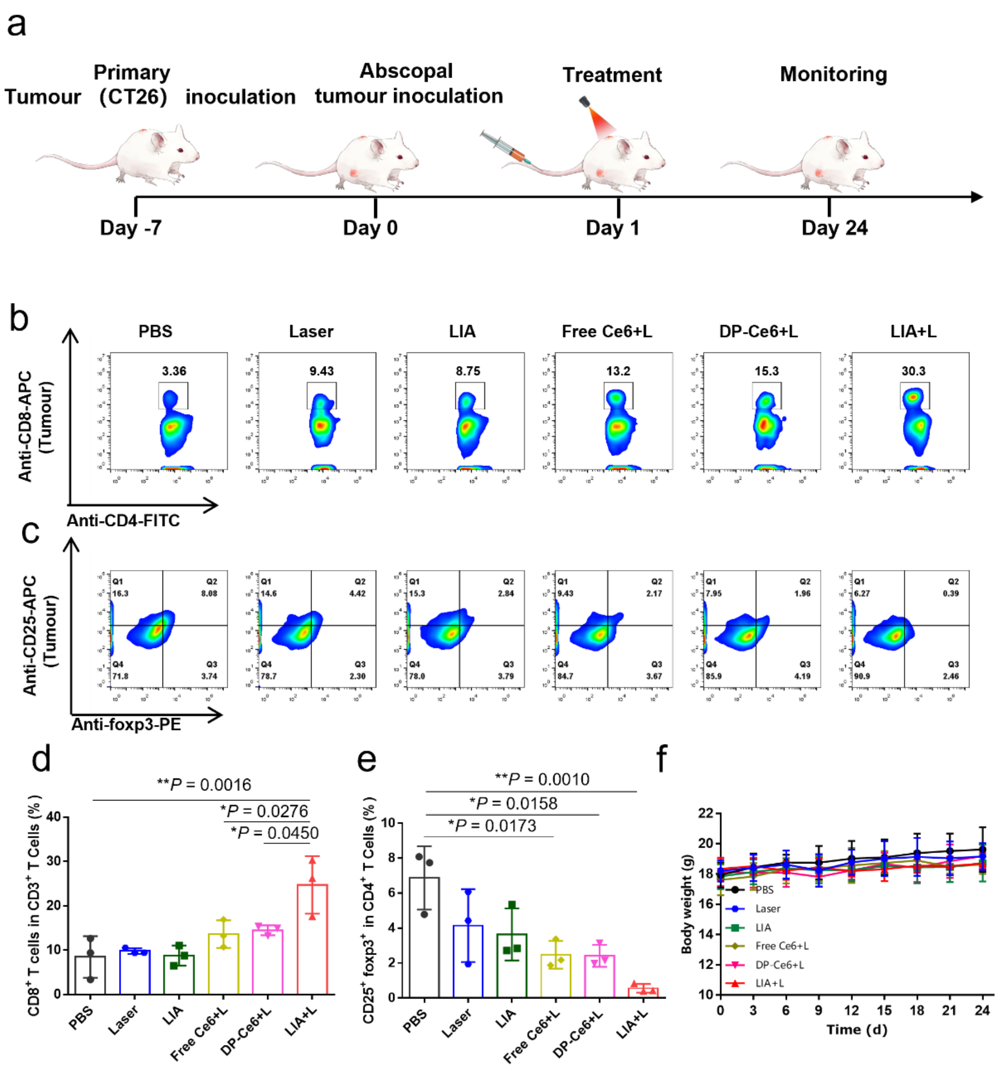

Supplementary Figure 22. Tumour inhibition in a CT26 bilateral tumour model. **a**, Schematic illustration of LIA-mediated antitumour efficency to inhibit primary tumour and abscopal tumour growth. b,c, Representative flow cytometry data of CD3$^+$ CD8$^+$ T cells in abscopal tumours (**b**) and CD4$^+$ CD25$^+$ foxp3$^+$ cell ratio in CD4$^+$ T cells (**c**). **d**,**e**, Statistical analysis of CD3$^+$ CD8$^+$ cell ratio in T cells (**d**), CD4$^+$ CD25$^+$ foxp3$^+$ cell ratio in CD4$^+$ T cells (**e**). **f**, Body weight of the CT26 tumour bearing BALB/c mice monitored during the anti-tumour therapy period. Data in (**d**,**e**) are presented as mean ± s.d. (n = 3), data in **f** are presented as mean ± s.d (n = 8). statistically significant differences between groups were identified by one-way ANOVA. ****$P < 0.0001$, ***$P < 0.001$, **$P < 0.01$, *$P < 0.05$.

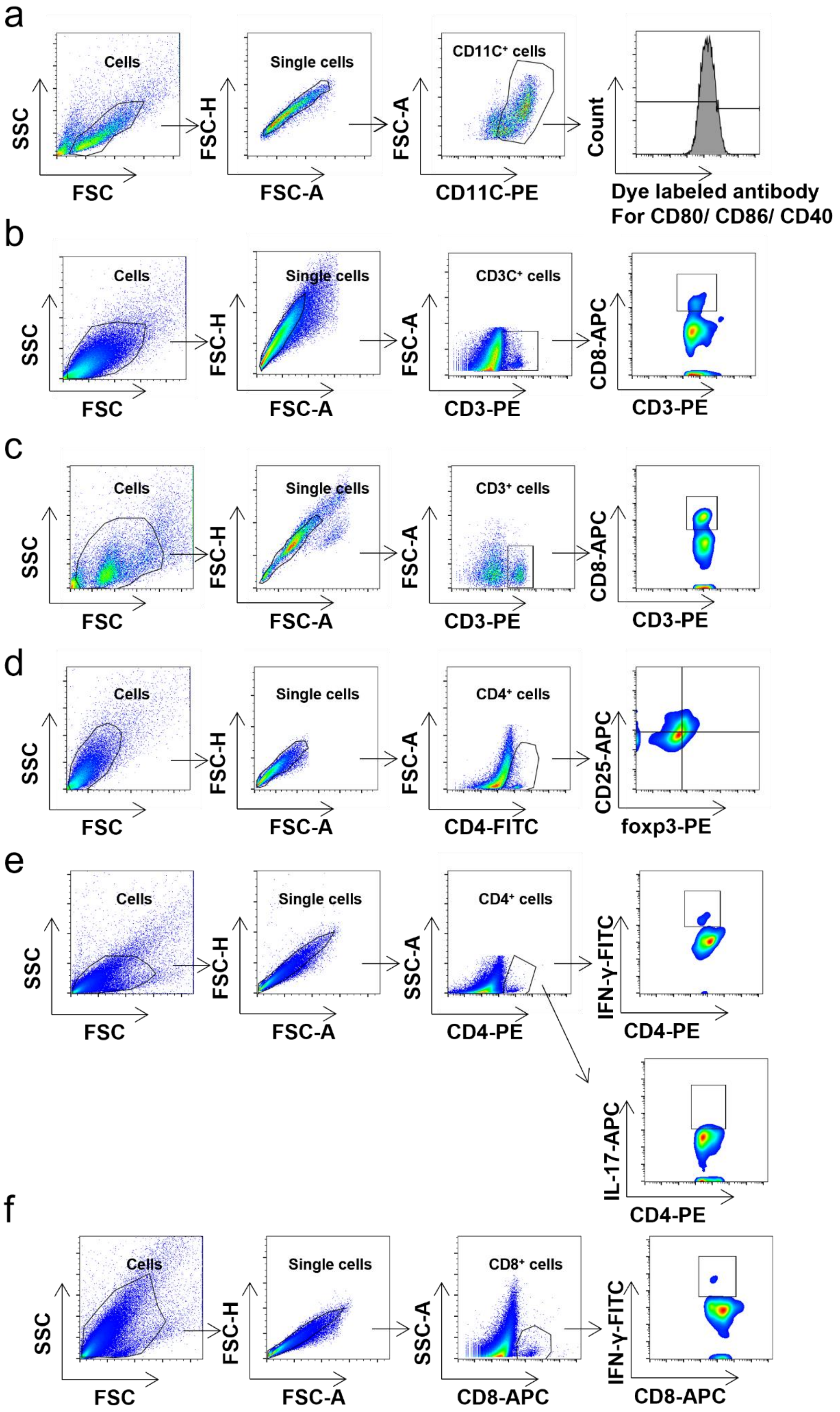
a
Cells
SSC
FSC
Single cells
FSC-H
FSC-A
CD11C⁺ cells
FSC-A
CD11C-PE
Count
Dye labeled antibody
For CD80/ CD86/ CD40
b
Cells
SSC
FSC
Single cells
FSC-H
FSC-A
CD3C⁺ cells
FSC-A
CD3-PE
CD8-APC
CD3-PE
c
Cells
SSC
FSC
Single cells
FSC-H
FSC-A
CD3⁺ cells
FSC-A
CD3-PE
CD8-APC
CD3-PE
d
Cells
SSC
FSC
Single cells
FSC-H
FSC-A
CD4⁺ cells
FSC-A
CD4-FITC
CD25-APC
foxp3-PE
e
Cells
SSC
FSC
Single cells
FSC-H
FSC-A
CD4⁺ cells
SSC-A
CD4-PE
IFN-γ-FITC
CD4-PE
IL-17-APC
CD4-PE
f
Cells
SSC
FSC
Single cells
FSC-H
FSC-A
CD8⁺ cells
SSC-A
CD8-APC
IFN-γ-FITC
CD8-APC

Supplementary Figure 23. Flow cytometry gating strategy for flow cytometry analysis. **a**, Flow cytometry gating strategy for the analysis of expression of CD80, CD86 and CD40 co-stimulators on DCs presented on Fig. 4a-f,j and Supplementary Figure 16a-f. **b**, Flow cytometry gating strategy for the analysis of $CD3^+$ $CD8^+$ T cells in tumour tissues presented on Fig. 6e,h and Supplementary Figure 22b,d. **c**, Flow cytometry gating strategy for the analysis of $CD3^+$ $CD8^+$ T cells in spleens presented on Fig. 5i,j and Fig. 6f,i. **d**, Flow cytometry gating strategy for the analysis of $CD4^+$ $CD25^+$ $foxp3^+$ T cells in tumour tissues presented on Fig. 6g,j and Supplementary Figure 22c,e. **e**, Flow cytometry gating strategy for the analysis of $CD4^+$ IFN-γ^+ T cells and $CD4^+$ IL-17^+ T cells in tumour tissues presented on Supplementary Figure 21b,c. **f**, Flow cytometry gating strategy for the analysis of $CD8^+$ IFN-γ^+ T cells in tumour tissues presented on Supplementary Figure 21d.

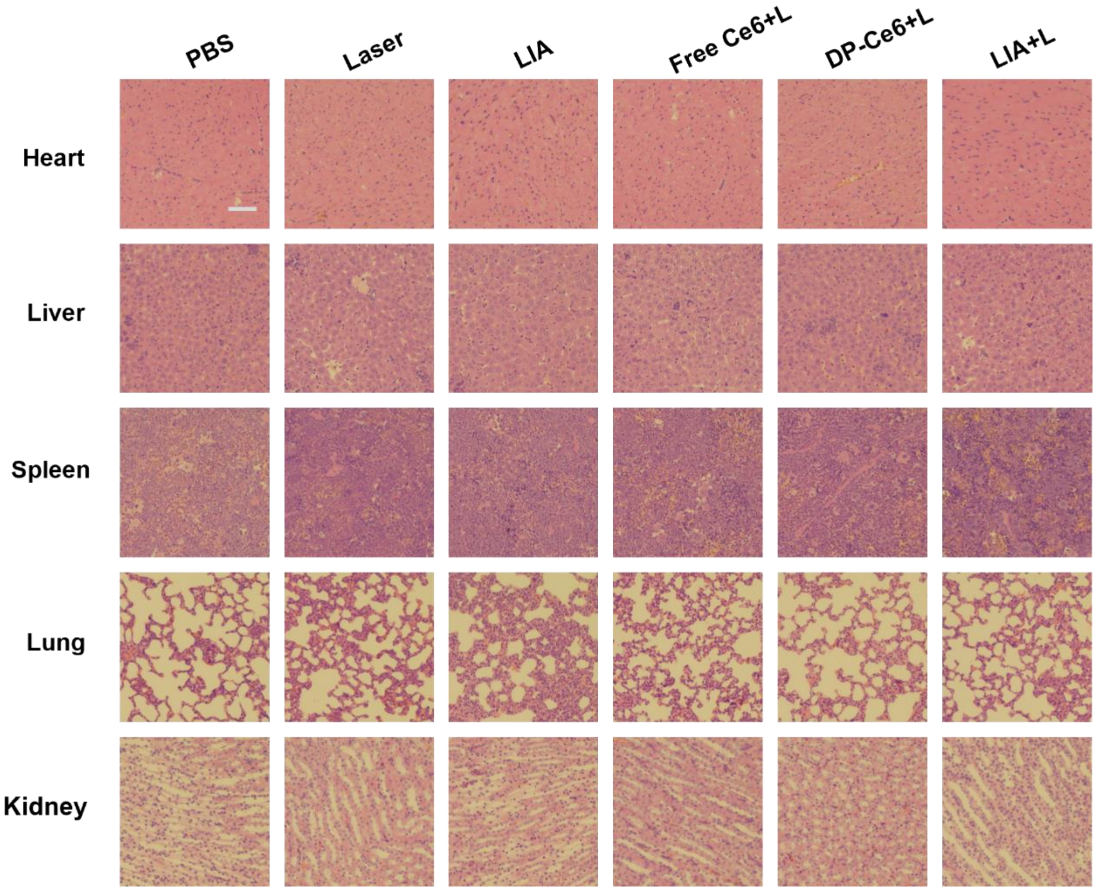

Supplementary Figure 24. Images of H&E staining of major organs of 4T1 bilateral tumour model. Mice were treated with different formulations. The major organs (heart, liver, spleen, lung and kidney) were collected at the end of treatments. Scale bar: 100 μm. Experiments were performed three times independently, representative images are shown.

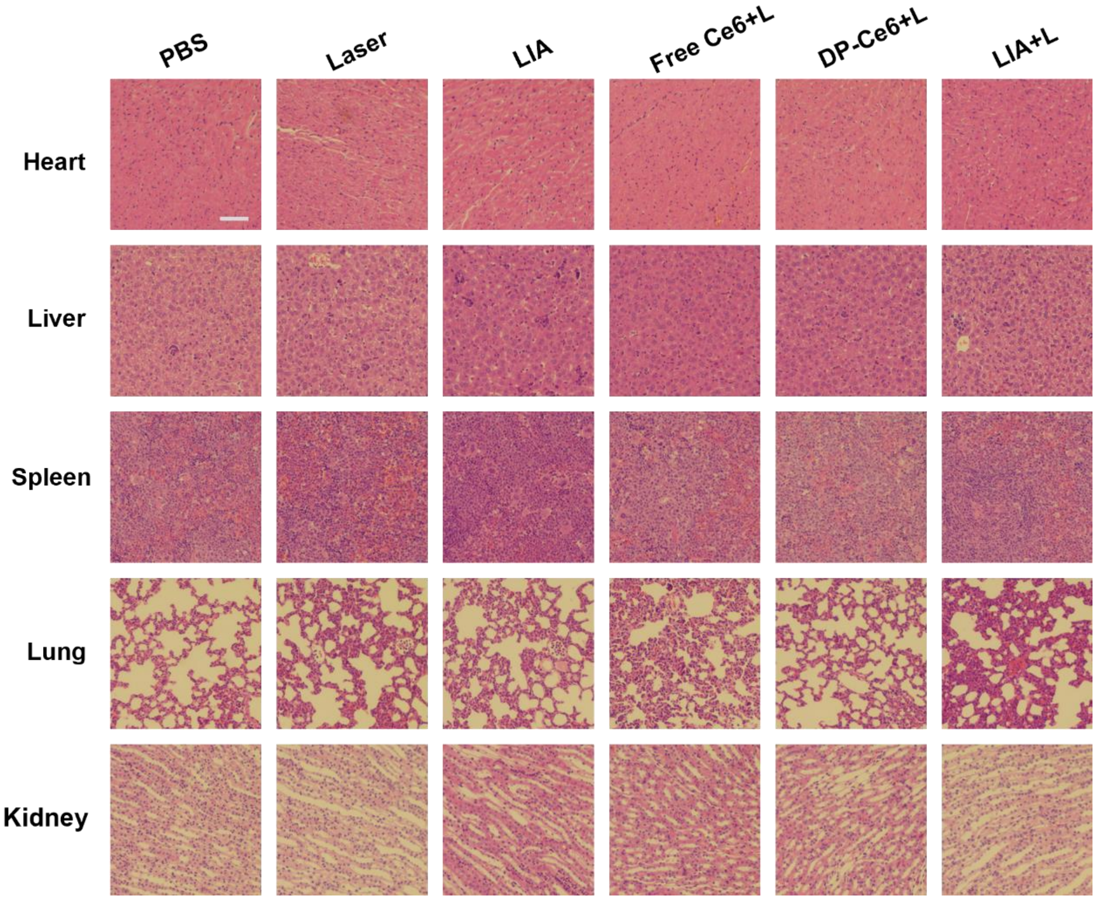

Supplementary Figure 25. Images of H&E staining of major organs of CT26 bilateral tumour model. Mice were treated with different formulations. The major organs (heart, liver, spleen, lung and kidney) were collected at the end of treatments. Scale bar: 100 μm. Experiments were performed three times independently, representative images are shown.

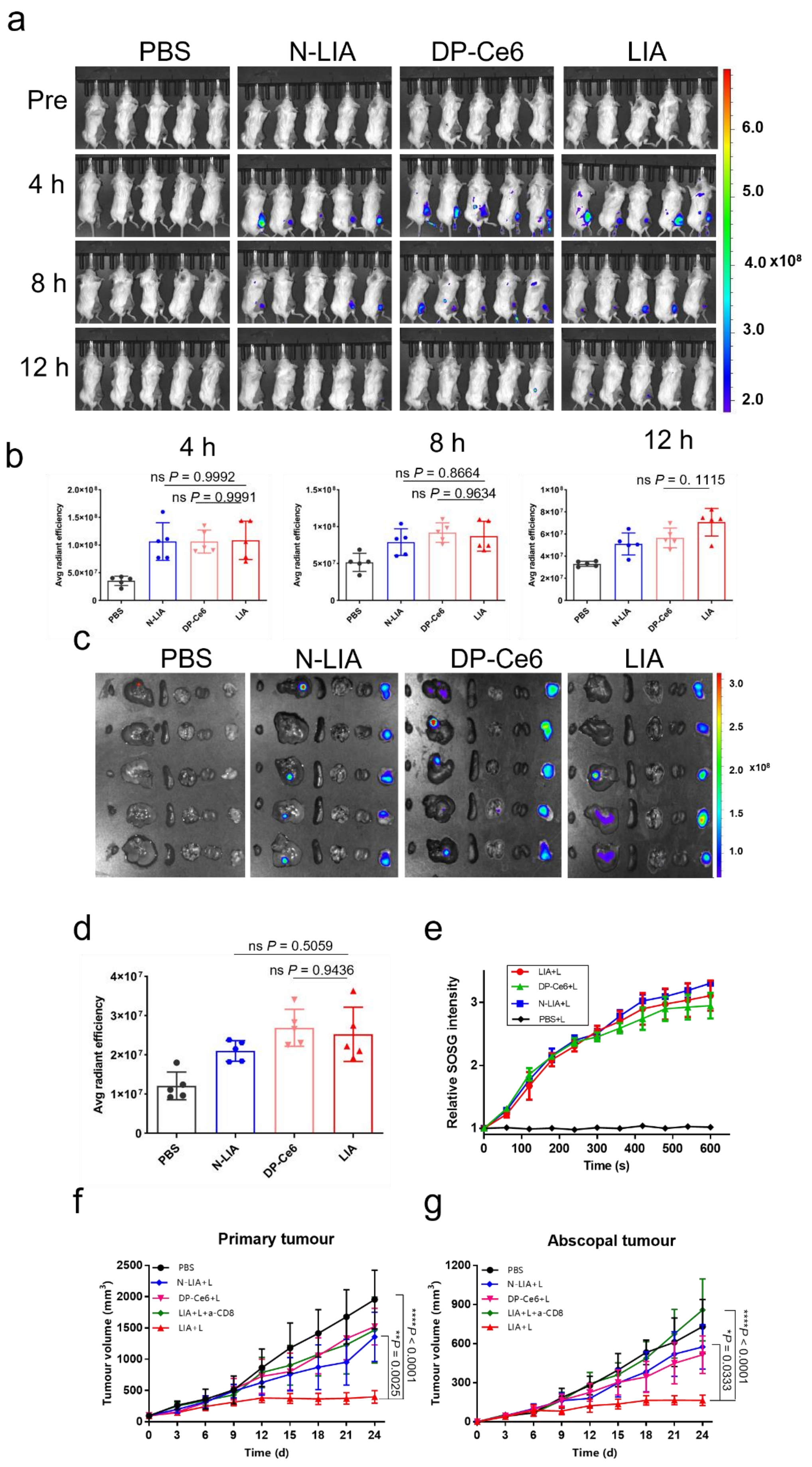
a
PBS
N-LIA
DP-Ce6
LIA
Pre
4 h
8 h
12 h
6.0
5.0
4.0 x10⁸
3.0
2.0
b
4 h
8 h
12 h
ns P = 0.9992
ns P = 0.9991
ns P = 0.8664
ns P = 0.9634
ns P = 0. 1115
Avg radiant efficiency
c
PBS
N-LIA
DP-Ce6
LIA
3.0
2.5
2.0 x10⁸
1.5
1.0
d
ns P = 0.5059
ns P = 0.9436
Avg radiant efficiency
PBS
N-LIA
DP-Ce6
LIA
e
LIA+L
DP-Ce6+L
N-LIA+L
PBS+L
Relative SOSG intensity
Time (s)
f
Primary tumour
Tumour volume (mm³)
PBS
N-LIA+L
DP-Ce6+L
LIA+L+a-CD8
LIA+L
Time (d)
****P < 0.0001
**P = 0.0025
g
Abscopal tumour
Tumour volume (mm³)
PBS
N-LIA+L
DP-Ce6+L
LIA+L+a-CD8
LIA+L
Time (d)
****P < 0.0001
*P = 0.0333

Supplementary Figure 26. Biodistribution, tumour accumulation and antitumour efficiency of different nanoparticles. **a**, Fluorescence images of the 4T1 tumor-bearing mice at 4, 8, 12 h post intravenous administration of N-LIA, DP-Ce6 and LIA at equivalent Ce6 dose (3 mg/kg). **b**, Quantification of mean fluorescence intensity of 4, 8, and 12 h post intravenous administration in a. **c**, Fluorescence images of major organs (heart, liver, spleen, lung, kidney) and tumor tissues collected at 24 h post injection, 5 mice per group. **d**, Quantification of mean fluorescence intensity of tumour tissues in c. **e**, Singlet oxygen generation abilities of N-LIA, DP-Ce6 or LIA (with equivalent Ce6 concentration of 10 ug/mL) were measured after the formulations were irradiated for different times. **f**,**g**, The antitumour efficacy of N-LIA, DP-Ce6 or LIA nanoparticles were evaluated in a bilateral tumor model. N-LIA, DP-Ce6 or LIA NPs were injected intravenously at equivalent Ce6 dose (3 mg/kg) and the tumor in the left flank (defined as primary tumor) was irradiated with NIR light 4 h post injection. The tumor in the right flank was defined as abscopal tumour. The treatments were performed three times every other day. For the $CD8^+$ T cells deletion group, the mice were pretreated with intraperitoneal injection of 100 μg of anti-CD8α monoclonal antibody on days 0 and 2. In the meantime, the mice were treated with LIA+L as described above. Tumour growth curves of primary tumours (**f**) and abscopal tumours (**g**) were evaluated (n = 5). Data in (**b**,**d**,**f**,**g**,) are presented as mean ± s.d. (n = 5), data in **e** are presented as mean ± s.d. (n = 3). Statistically significant differences between groups were identified by one-way ANOVA. ****P < 0.0001, ***P < 0.001, **P < 0.01, *P < 0.05.

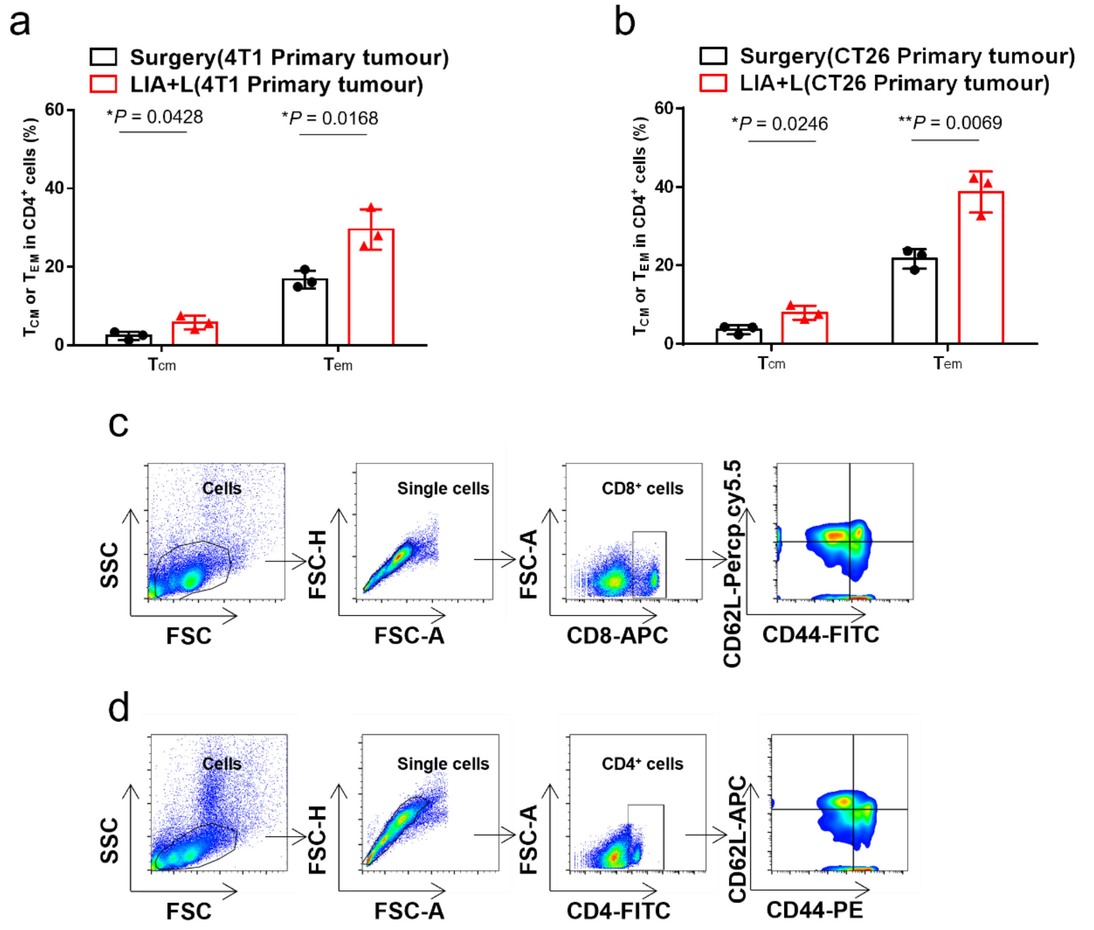

Supplementary Figure 27. Analysis of memory T cells in CD4$^+$ T cells and flow cytometry gating strategy for the analysis of memory T cells. **a**,**b**, Statistical analysis of CD3$^+$CD8$^+$CD44$^+$CD62L$^+$ (T_{CM}) cell or CD3$^+$CD8$^+$CD44$^+$CD62L$^-$ (T_{EM}) cell percentages in CD4$^+$ cells in 4T1 tumour (**a**) and CT26 tumour (**b**) model 30 days after different treatments. c,d, flow cytometry gating strategy for the analysis of memory T cells in CD8$^+$ T cells (**c**) and CD4$^+$ T cells (**d**). Data in (**a**,**b**) are presented as mean ± s.d. (n = 3). Statistically significant differences between groups were identified by two-tailed unpaired Student's t-test. ****P < 0.0001, ***P < 0.001, **P < 0.01, *P < 0.05.

Supplementary Table 1. Dynamic molecular docking of HAD and rHAD head groups with TLR7. Simulated calculations of the interaction affinity by the Grid scoring function.

Group	PDB Code	Grid score（kcal/mol）	Grid-vdw（kcal/mol）	Grid-es（kcal/mol）	Internal Energy（kcal/mol）
HAD	5mgh	-48.44	-45.19	-3.25	5.33
rHAD	5mgh	-51.08	-43.28	-7.80	7.51

Supplementary Table 2. Characterizations of the prepared nanoparticles. The size of nanoparticles was measured with DLS. Ce6 encapsulation efficiency and drug loading in the particles were measured using Uv-vis spectrophotometry. Data are presented as mean ± s.d. (n = 3) from three independent experiments.

NPs	DP-Ce6	N-LIA	LIA
Size (nm)	32.77 ± 3.9	47.49 ± 2.8	43.22 ± 3.4
Encapsulation efficiency	89.7 ± 4.1 %	81.3 ± 1.9 %	82.4 ± 3.0 %
Drug loading	12.7 ± 0.7 %	13.4 ± 0.4 %	14.1 ± 0.4 %
